# New Frontiers in Contrast-Enhanced Ultrasound for Cancer Imaging

**DOI:** 10.1021/acsnano.5c17992

**Published:** 2026-05-12

**Authors:** Felipe Matias Berg, Michaela Briana Cooley, Theresa Kosmides, Laura E. Chen, Ronaldo Hueb Baroni, Agata A. Exner

**Affiliations:** † Department of Radiology, 2546 Case Western Reserve University, Cleveland, Ohio 44106, United States; ‡ Department of Radiology, 37896 Hospital Israelita Albert Einstein, 05652-900 São Paulo, SP, Brazil; § Department of Biomedical Engineering, 2546 Case Western Reserve University, Cleveland, Ohio 44106, United States

**Keywords:** nanobubbles, contrast media, ultrasonography, neoplasms, molecular imaging, nanoparticles, interventional radiology, microbubbles, early diagnosis, multimodal imaging

## Abstract

Nanobubbles (NBs) are increasingly recognized as the next generation of ultrasound contrast agents that can provide alternate avenues for the advancement of cancer imaging and therapy. Compared to currently clinically viable microbubbles that are limited to the vasculature because of their size, nanobubbles with diameters ranging from 200 to 500 nm can potentially target the perivascular and possibly extravascular compartments. Their polymeric, lipid, or hybrid shells can be engineered to tune stability, increase circulation time, and support surface functionalization with ligands to facilitate receptor-directed binding. Preclinical studies show that NBs can extend ultrasound imaging windows, improve sensitivity, and increase tumor-to-background contrast across different types of cancers. NBs can also be formulated with secondary reporters that allow their combination with other imaging modalities, such as optical (photoacoustic and fluorescence) and magnetic resonance imaging. However, several challenges need to be overcome to allow the clinical translation of NBs, such as acoustic optimization and standardization, formulation and manufacturing reproducibility, and comprehensive safety characterization. Addressing these barriers will be essential to establishing NBs as clinically viable agents. Here, we summarize recent advances in NB design and functionalization, review key preclinical oncology applications, and discuss translational priorities to support their integration into precision oncology clinical workflows.

## Graphical abstract

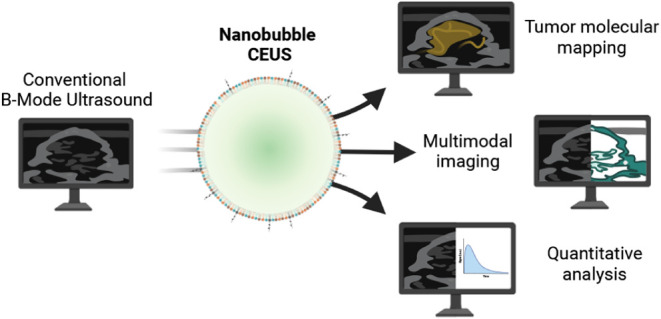

## 1 Introduction

Cancer remains one of the most important challenges in global healthcare, even with major advances in diagnosis and treatment over the past few decades. An estimated 10 million cancer-related deaths occur each year, leading to not only stress over the healthcare system but also an extraordinary economic burden as well.^1–4^ Projections based on macroeconomic models indicate that the costs to the economy as a result of the advent of cancer between the years 2020 and 2050 are set to exceed a whopping US $25 trillion.^5^ The effects in terms of both expenditure and health disparities do not fall evenly across regions, while early diagnosis and immediate treatment are core to the successful reduction of mortalities due to cancer and the overall financial costs, about 70% of total deaths due to cancer are in areas where proper screening is rarely implemented, even in the presence of valid screening methods.^6^ In addition, while there can be early diagnosis of the diseases, curative interventions in these conditions entail highly resource-consuming and risky surgeries, which in turn are applicable only in selected locations.^7,8^ Together, these gaps highlight the need for more accessible diagnostic and therapeutic platforms for cancer.

Ultrasound is uniquely positioned to meet this need because of its availability worldwide, comparatively low-cost, and capability of point-of-care real-time imaging.^9,10^ Yet in oncology, conventional ultrasound underperforms in terms of lesion characterization and comprehensive disease assessment—limitations that are driven in large part by the contrast paradigm rather than the imaging platform itself.^11^ Contrast-enhanced ultrasound (CEUS), which combines intravenous administration of gas-filled microbubble (MB) agents with contrast-specific imaging sequences, enables real-time visualization of tumor perfusion and vascular phenotype, improving focal lesion characterization, early response assessment, and dynamic guidance for image-directed interventions.^12,13^ However, clinical oncologic CEUS remains largely centered on focal liver lesion characterization, which is currently the only Food and Drug Administration (FDA)-approved oncologic indication for intravenous CEUS in the United States.^14,15^ Beyond the liver, CEUS is increasingly used in renal oncology for the surveillance of indeterminate renal masses and for post-treatment assessment following nephron-sparing therapies.^16,17^

Despite their established safety and utility, clinically used MBs are fundamentally constrained by their size and pharmacokinetics. With diameters on the order of 1–10 μm, they remain confined to the vascular space and provide only transient blood-pool enhancement, limiting their ability to interrogate extravascular hallmarks of cancer such as receptor expression within the tumor microenvironment, stromal architecture, or heterogeneous viable vs necrotic regions beyond perfused vessels.^18,19^ As a result, MB-based CEUS is highly effective for assessing vascularity and perfusion dynamics,^20^ but it is less suited to the emerging “new frontiers” in oncologic imaging—namely, robust molecular imaging beyond the blood pool, longer-lasting and more controllable signals for longitudinal or whole-tumor assessment, and integration with therapeutic functions such as ultrasound-triggered delivery, vascular modulation, or direct mechanical bioeffects.^21^ Addressing these next-step goals requires contrast agents engineered to access and report on tumor biology beyond the intravascular compartment while retaining the real-time, bedside advantages that make ultrasound clinically attractive.

Nanobubbles (NBs) are submicron, gas-core ultrasound contrast agents (UCAs) engineered to overcome the intrinsic blood-pool constraints of MBs and thereby expand what CEUS can report in oncology.^22–24^ As such trends move forward, the number of patents related to NBs and the number of PubMed-listed articles related to the use of medical ultrasound have considerably increased (Figure 1). The search strategies used to generate these patent and publication trends are provided in the Supporting Information, which is available online. By tuning size and surface chemistry, NBs can achieve altered pharmacokinetics and longer-lasting signals than conventional agents, facilitating more sustained assessment of tumor perfusion and spatial heterogeneity. In addition, ligand-functionalized shells enable molecularly targeted CEUS, providing receptor-specific enhancement that extends ultrasound imaging beyond generic blood-pool contrast toward biologically informed characterization.^22,24–27^ Although extravasation is formulation- and tumor-dependent, the submicron scale may also permit access to perivascular and tumor microenvironment regions that are largely inaccessible to micron-sized agents.^23,28,29^ Finally, NBs provide a platform for ultrasound-triggered therapeutic functions, including triggered release of payloads, vascular modulation, and cavitation-mediated bioeffects, positioning them as promising theranostic agents that couple real-time imaging with on-demand intervention.^25,30^ These capabilities are governed by key design parameters—including shell composition (e.g., lipid vs polymer architectures, PEGylation/stabilizers for circulation and stealth), gas core selection (e.g., perfluorocarbon gases for stability), and surface functionalization (targeting ligands and responsive motifs)—which together dictate stability, acoustic response, biodistribution, and ultimately clinical translatability.^23,31,32^

**Figure 1:**
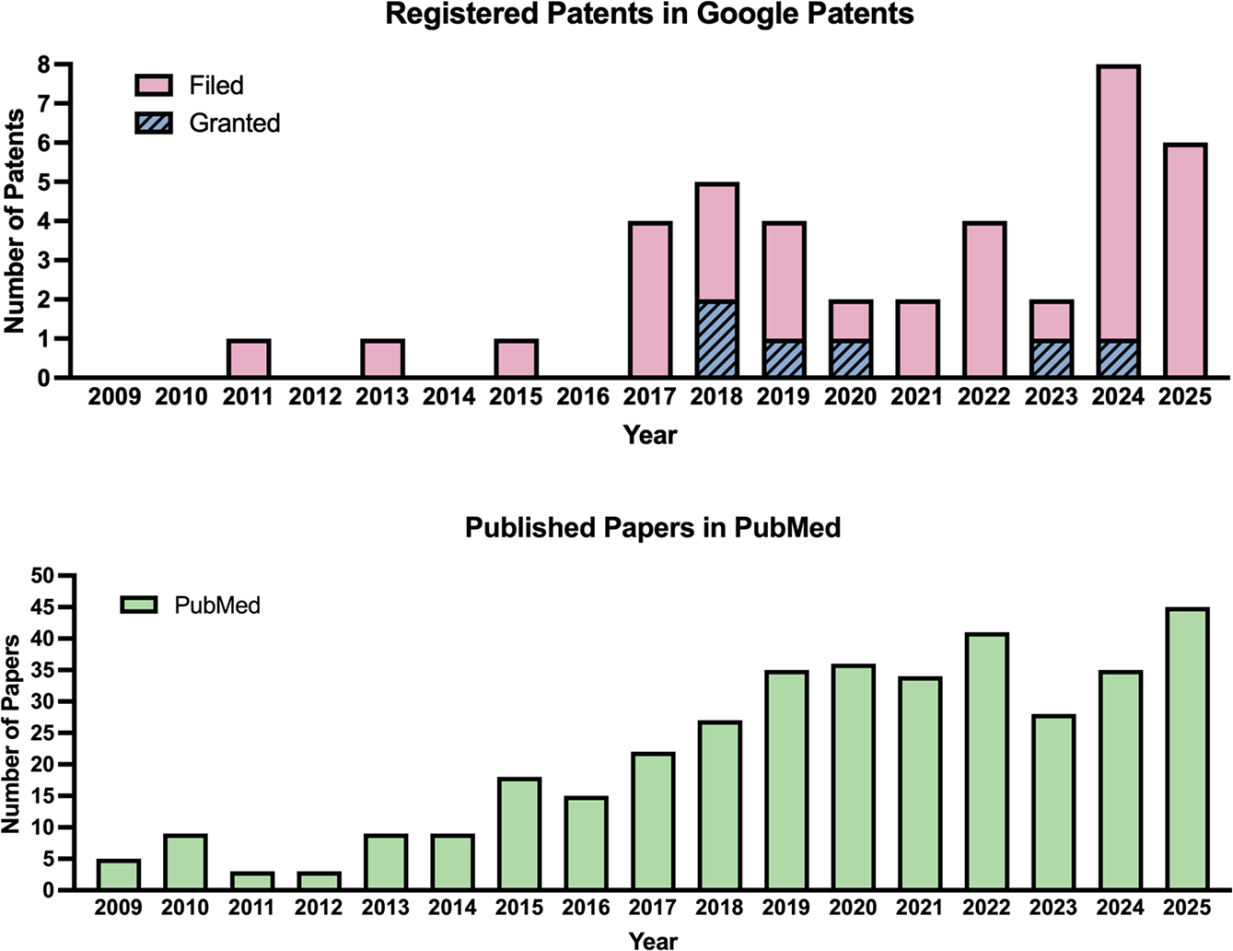
Trajectory of NB UCA research and intellectual property. The numbers of (A) NB patents filed/granted and (B) PubMed publications related to NB for medical or ultrasound-related topics over the years. The search strategy is provided in the Supporting Information File.

In this review, we first summarize the physicochemical design principles that govern NB stability, acoustic response, and *in vivo* behavior, and then synthesize the state of the art in oncologic imaging applications, spanning perfusion and functional imaging, molecularly targeted imaging, and emerging approaches for quantitative mapping and treatment-response assessment. We next discuss multimodal strategies and other extensions enabled by NB formulations (e.g., optical and photoacoustic add-ons) and conclude by outlining the key technical and translational barriers—from manufacturing and characterization standards to acoustic performance and reproducibility, safety evaluation, and regulatory considerations—that must be addressed for clinical adoption.

## 2 Ultrasound

Since its first oncologic application for breast tumors reported in 1952 by Wild and Reid,^33^ ultrasound imaging has come a long way and is currently an essential part of modern oncology care.^34^ The widespread adoption of ultrasound imaging can be attributed to its ease of use, safety (lack of ionizing radiation), and cost-effectiveness compared with other imaging modalities. Ultrasound imaging also offers specific economic and infrastructural benefits, as it is about five or six times less costly compared to MRI and there is no need for specific premises, allowing for its use in operating rooms or in areas where there is a lack of infrastructure. In terms of its application, ultrasound imaging is used in a wide range of oncologic settings,^9,10^ ranging from the monitoring of high-risk individuals for hepatocellular carcinoma^35^ to its primary use as a first imaging step for percutaneous image-guided biopsy of the prostate, lymph nodes, thyroid, and other organs.^36^ Ultrasound imaging also serves as a powerful tool for oncology-related applications, including treatment planning,^37^ therapy monitoring,^38^ and post-treatment surveillance,^39^ performing as a versatile platform for oncology applications.

### 2.1 Emerging Technologies in Clinical Ultrasound Imaging

Emerging innovations in ultrasound technology are constantly increasing its potential for diagnosis and treatment. High-frequency microultrasound (≈29 MHz), for example, has shown equal efficacy to MRI-ultrasound fusion for guiding prostate biopsy procedures.^40^ Shear wave elastography has also been successfully employed for prostate cancer diagnosis, with sensitivity and specificity ranging from 84% for distinguishing malignant from normal tissues.^41^ At a microvascular scale, super-resolution ultrasound has been shown to be effective in visualizing vessels as small as 100 μm, thus providing a way to study tumor-specific microvascular architecture and assess changes resulting from treatment.^42^ On the therapeutic front, noninvasive modalities such as high-intensity focused ultrasound (HIFU) have achieved biochemical failure-free survival rates of approximately 70% in prostate cancer,^43^ while histotripsy shows over 95% technical success in the ablation of liver tumors without the need for incisions.^44^

### 2.2 Ultrasound Contrast Agents

UCAs are gas-filled MBs encapsulated by lipid, protein, or polymer shells^45^ and a gas core—commonly air, perfluoropropane (C_3_F_8_), or sulfur hexafluoride (SF_6_).^46^ When insonated, MBs oscillate nonlinearly and produce strong contrast signals because the gas core is acoustically mismatched with surrounding tissues.^46–48^ Due to their size (1–10 μm), clinically approved UCAs (Table 1) are restricted to the vascular space and cannot extravasate into the interstitial compartment.^49^ UCAs are highly safe, with adverse events—primarily hypersensitivity or vasovagal reactions—occurring in fewer than 0.01% of administrations.^50^

**Table 1:** FDA-Approved UCAs^a^

proprietary name	particle size (μm)	gas core	shell	labeler name	approval	application
Optison	3–4.5	C_3_F_8_	albumin	GE Healthcare	1997	echocardiography^51^
Definity	1.1–3.3	C_3_F_8_	phospholipid, PEG, PG	Lantheus Medical Imaging	2001	echocardiography^52^
Lumason	1.5–2.5	SF_6_	phospholipid and PEG	Bracco Diagnostics	2014	echocardiography
						liver lesion characterization
						pediatric vesicoureteral reflux evaluation^53^
Definity RT	1.1–3.3	C_3_F_8_	phospholipid, PEG, PG	Lantheus Medical Imaging	2020	echocardiography^54^

a FDA-approved ultrasound contrast agents as of 2025. Mean particle diameter and application per prescribing information. C_3_F_8_, perfluoropropane; SF_6_, sulfur hexafluoride; PEG, polyethylene glycol; PG, propylene glycol.

#### 2.2.1 Clinical Uses of Contrast-Enhanced Ultrasound

Despite the rapidly expanding applications of CEUS, regulatory approval of MB agents remains largely confined to cardiac imaging, with the sole exception of Lumason MB, which is also indicated for liver lesion characterization.^53^ In this setting, Lumason MB-enhanced CEUS achieves a sensitivity of approximately 95% and a specificity of 94% for distinguishing malignant from benign hepatic lesions^14,15,55^ and it outperforms computed tomography (CT) and MRI in post-transarterial chemoembolization (TACE) assessment of hepatocellular carcinoma (HCC), with a 91% sensitivity for detecting residual viable tumor.^56^ This performance is attributable to the hypervascularity of HCC—MBs remain strictly intravascular, thereby delineating the rapid wash-in and wash-out kinetics of tumor neovasculature relative to normal parenchyma.^57,58^ Nonetheless, the strictly intravascular nature of MBs confines their role to vascular imaging, limiting their broader oncologic utility and prompting investigation of novel MB formulations in clinical trials.^23^

#### 2.2.2 Clinical Evaluation of Microbubble Contrast Agents in Cancer

Over the current decade, the number of CEUS-registered clinical trials has more than doubled relative to the prior decade (151 since 2016 vs 72 between 2006 and 2015), including 74 ongoing studies as of November 2025.^59^ This is due in large part to the growing use of CEUS as a complementary discipline within conventional oncologic imaging and image-guided interventions, as well as its relevance as a platform technology for novel applications of ultrasound.

While this expansion highlights increasing interest in CEUS, its translation into clinical practice for MB UCAs other than for liver diagnostics has been inconsistent. In breast cancer, Lumason has been tested for sentinel lymph node detection, but with limited efficacy, averaging only 1.3 nodes identified—well below the standard methods such as blue dye and the guideline-recommended minimum of two nodes.^60^ In thyroid cancer, sentinel lymph node detection using Lumason achieved detection rates above 90%, yet false negatives persisted due to impaired lymphatic drainage or nodal tumor burden.^61^ Recently, new agents designed for molecular imaging have entered clinical trials. BR55 (Bracco Diagnostics, Italy), a VEGFR2-targeted agent, established a correlation between contrast-enhanced ultrasound (CEUS) enhancement and immunohistochemical expression of kinase insert domain receptor (KDR) in breast and ovarian cancers.^62,63^ However, as BR55 targets angiogenesis rather than tumor-specific markers, its sensitivity was found to be low, especially for indolent diseases like prostate cancer, as low as 50%.^64^

## 3 Nanobubbles

### 3.1 Structural Components of Nanobubbles

NBs, also referred to as ultrafine bubbles, are submicron (<1 μm) gas-filled, shell-stabilized structures designed to serve as ultrasound-responsive contrast agents and drug delivery vehicles (Figure 2).^22^ From a materials perspective, their performance reflects coupled core–shell properties—gas composition, shell permeability/viscoelasticity, and surface chemistry—that jointly govern stability, acoustic activity, and biological interactions. As with commercial MBs, their core typically contains biologically inert gases such as perfluoropropane (C_3_F_8_) or sulfur hexafluoride (SF_6_), selected for their low solubility and diffusivity in plasma.^65^ While chemical inertness and established use in FDA-approved UCAs have been requirements, these gases are also characterized by a large acoustic impedance mismatch against biological soft tissues and bodily fluids, thereby allowing second-harmonic generation in response to pressure changes from ultrasound and, consequently, improved echogenic properties.^65^ At nanoscale sizes, retaining the encapsulated gas becomes increasingly sensitive to the shell’s barrier and mechanical properties: if the shell has even modest permeability (or insufficient viscoelastic resistance), the high Laplace pressure at the nanoscale can markedly accelerate gas diffusion and bubble dissolution unless the formulation is specifically optimized. As expected, based on these considerations, both intensity and pressure-dependent properties of this harmonic signal generation become similarly dependent on the viscoelastic properties of bubble shells, as well as the size variance of NBs, to provide similar reproducibility of imaging properties.

**Figure 2:**
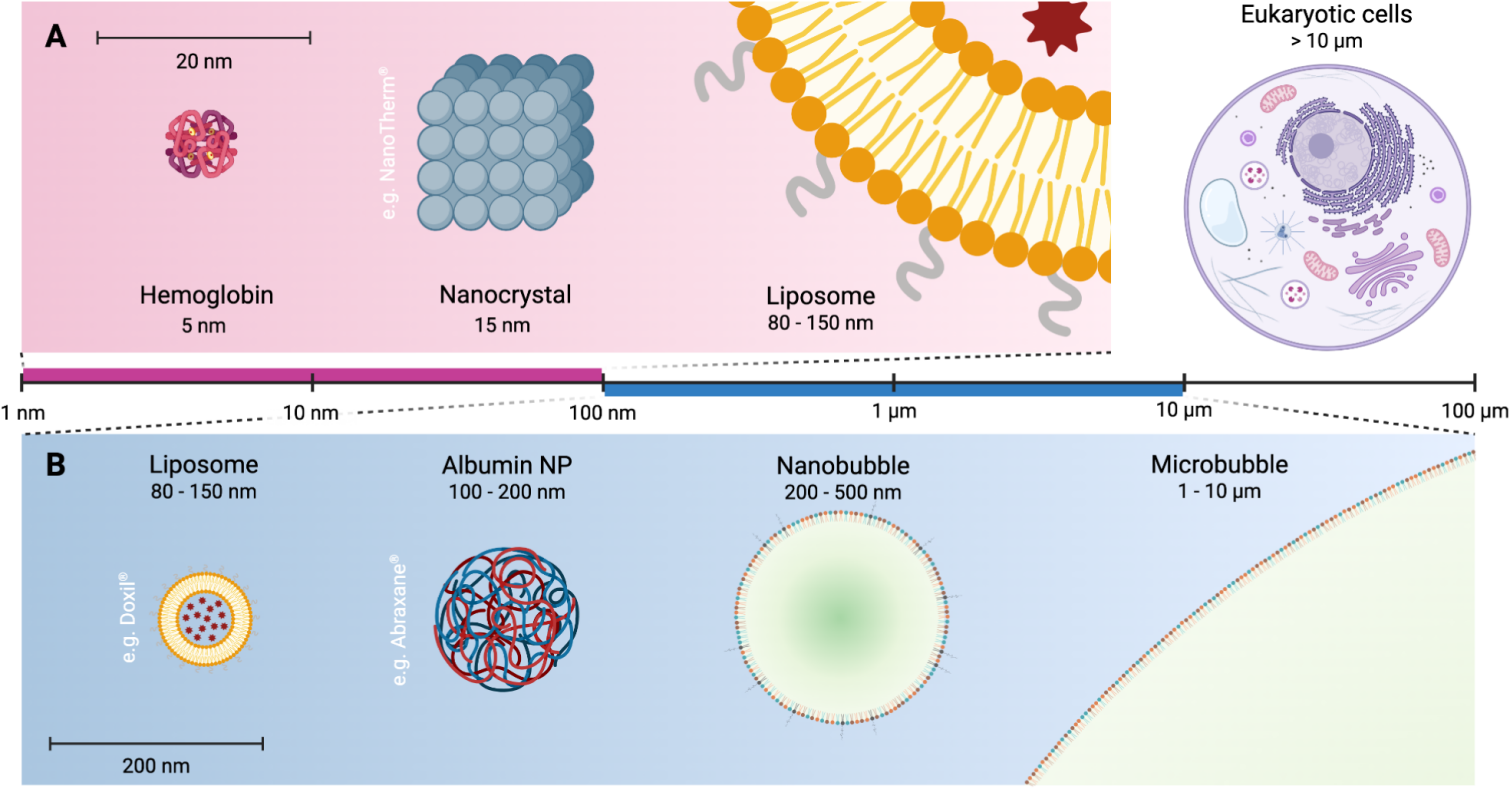
Comparative size distribution of NBs and FDA-approved nanomedicine platforms used in cancer applications. Panels A and B show size comparison across nanoparticles on a linear scale. NP: nanoparticle. Made with BioRender.

The gas core is surrounded by a stabilizing shell that is often made from phospholipids, such as dipalmitoylphosphatidylcholine (DPPC), distearoylphosphatidylglycerol (DSPG), or dipalmitoylphosphatidic acid (DPPA). This shell composition can be adjusted to tune the surface charge, stiffness, and circulation time (Figure 3).^66,67^ For instance, many formulations include PEGylated lipids, such as DSPE-PEG2000, to reduce aggregation and prolong circulation. PEG provides a steric barrier that limits protein adsorption and clearance by the mononuclear phagocyte system and also offers a convenient handle for attaching targeting ligands.^68^ Other shell choices consist of biodegradable polymers like PLGA or chitosan, which can increase robustness and are often advantageous in terms of drug loading and release.^69,70^ Other cosolvents like glycerol and propylene glycol are also frequently added to the formulation to control the viscosity and elasticity of the shell, which could be advantageous in terms of *in vivo* persistence and signal.^31^ All these formulation choices are associated with trade-offs, where a stiffer and less permeable shell increases robustness and persistence at the cost of oscillation and nonlinear scattering, while a soft shell has the opposite effect.

**Figure 3:**
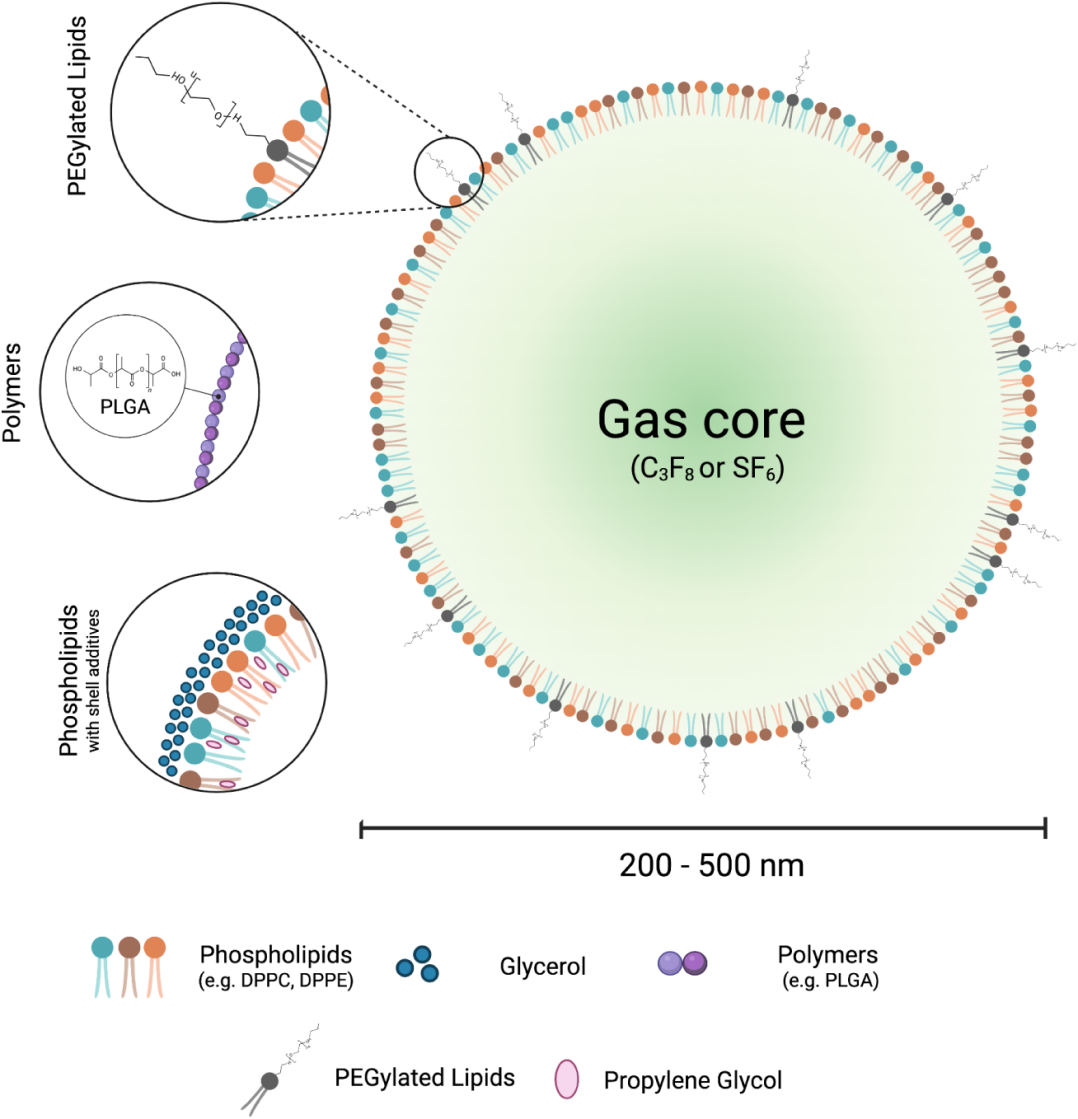
Schematic representation of a nanoscale ultrasound contrast agent (NB) and its different possible shell compositions. Made with BioRender.

### 3.2 Formulation and Characterization

Several methods have been developed in the fabrication of nanobubbles (NBs), and the majority of the techniques involve lipid film hydration and mechanical stirring followed by differential centrifugation for the separation of submicrometer-sized particles.^31^ Recently, microfluidic-based fabrication and membrane extrusion techniques have been explored for the fabrication of nanobubbles in order to obtain a more uniform size distribution and improve the reproducibility of the process while minimizing the amount of material required and maximizing the yield.^71,72^ While the theoretical basis of the process has been established, the process is highly dependent on the technique adopted. Hence, it may be emphasized that fabrication is a crucial aspect of the process rather than a minor aspect of the technique. In this regard, a comparison of the techniques has been made by Wegierak et al.^22^

The characterization of NBs is a complex challenge because of their small size, compressibility, and gaseous content. Resonant mass measurement (RMM) is one of the few techniques that can distinguish buoyant NBs from nonbuoyant particles, which is a considerable advantage over optical particle size analysis.^73^ Other techniques, such as dynamic light scattering (DLS) and nanoparticle tracking analysis (NTA), are commonly used to determine the hydrodynamic size of particles based on Brownian motion. However, these techniques are biased toward larger particles and are challenged by NBs because of their low refractive index.^74,75^ Moreover, although NTA allows the tracking of individual particles and the measurement of particle concentrations, the ability to distinguish NBs from other particles is poor.^74,75^

A central issue is that even a small micron-scale contaminating fraction can dominate the acoustic signal. As a result, size measurement alone is not enough—bubble identity and gas-core composition should be confirmed with orthogonal measurements. Coulter-based methods are generally not well suited for NBs because compressible, fragile bubbles displace little electrolyte and can deform or rupture during measurement.^76^ Finally, gas chromatography/mass spectrometry (GC/MS) can verify core gas identity and encapsulation efficiency and is often used to assess residual solvents.^76^ Together, these constraints have contributed to inconsistent “NB” definitions across the literature and can complicate comparisons between studies. For that reason, Section 5.1 outlines minimum orthogonal validation and reporting standards to improve comparability and reduce misattribution.

### 3.3 Functionalization

Functionalizing NBs allows researchers to add targeting ligands, imaging reporters, therapeutic payloads, or even other nanocarriers to or within the bubble. These additions broaden the scope of NBs from molecular imaging and hybrid imaging readouts to drug and nucleic acid delivery. While strategies for therapeutic functionalization have been extensively reviewed by Chen et al.,^23^ here, we focus on approaches most relevant to cancer imaging.

A common strategy is utilizing standard functionalization protocols to anchor ligands through PEGylated lipids, which is straightforward and works well with NB self-assembly.^28^ Ligands such as peptides and small molecules (e.g., PSMA-1) can be incorporated during formulation with minimal disruption to the shell.^28^ For covalent attachment, EDC/NHS chemistry can be applied to both lipid- and polymer-based shells and supports the conjugation of a wide range of biomolecules, including antibodies, peptides, and aptamers.^77^ Thiol–maleimide coupling offers more site-specific attachment through engineered thiols, which can be especially useful for proteins and thiolated peptides.^78^ Biotin–streptavidin binding is often used in modular designs because of its very strong affinity and ease of assembly, including multivalent formats with antibodies or nanobodies.^26^ Lastly, physical entrapment is another noncovalent method that is useful for the loading of hydrophobic dyes or small molecules.^79^ Other strategies are also used for conjugation, although these are the most common ones in the field. Figure 4 shows a summary of the most commonly used functionalization strategies for the engineering of NBs.

**Figure 4:**
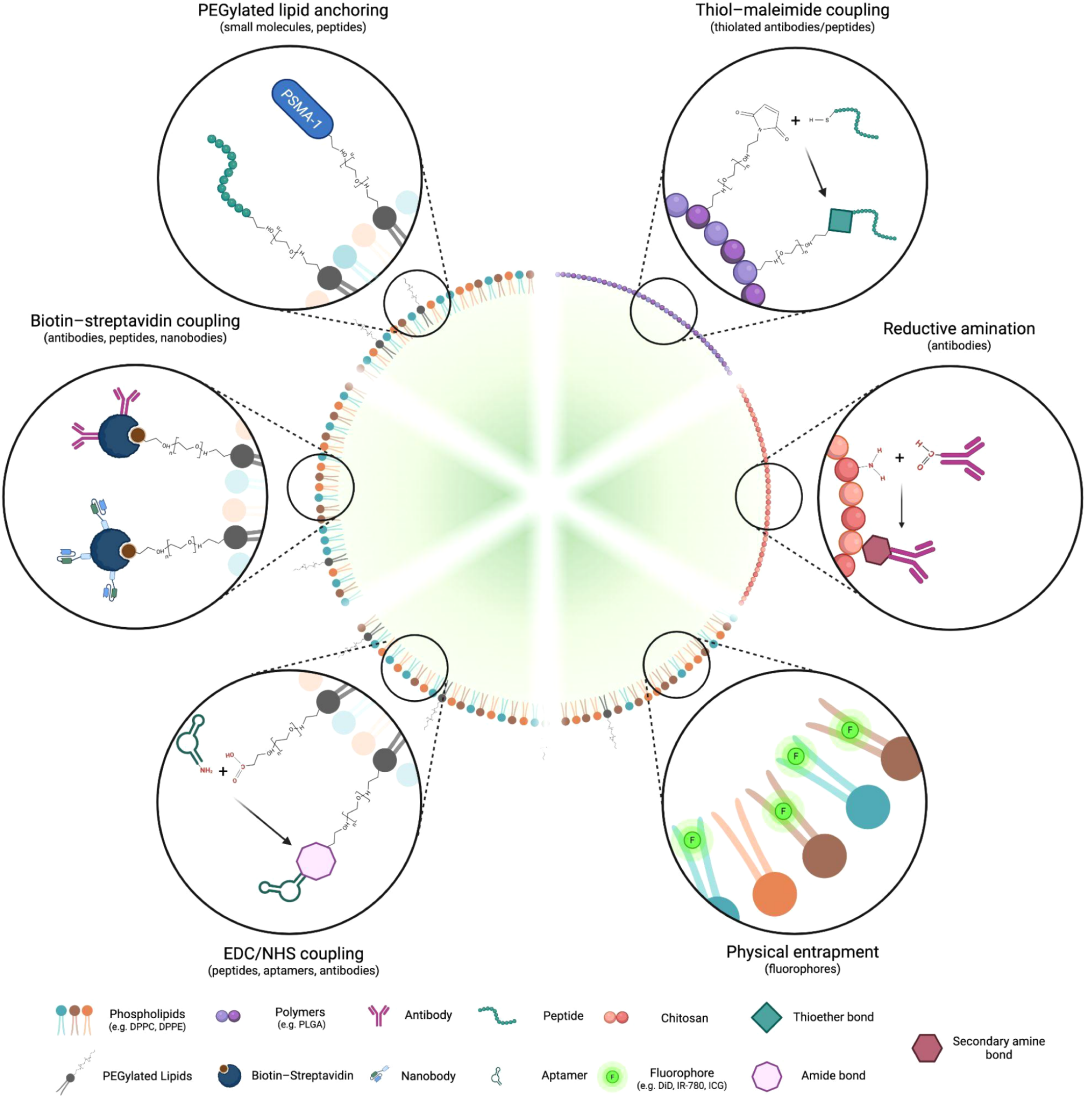
Schematic representation of major ligand integration strategies used for NB shell functionalization. Made with BioRender.

### 3.4 Acoustic Properties

NBs, like other gas-based UCAs, are thought to enhance ultrasound signals through nonlinear behavior in response to an increased pressure. While tissue signals are linear, NBs are thought to produce harmonic, subharmonic, and ultraharmonic signals in response to 50 to 400 kPa. These signals are thought to be obtained using specific pulse sequences such as pulse inversion or pulse amplitude modulation.^80,81^

Due to their small size, NBs are thought to produce signals at a higher frequency than MBs. Because a 2 μm diameter MB has a resonant frequency of 3.8 MHz, it is expected that NBs will not be able to produce signals at this frequency. However, despite their higher theoretical resonance frequencies, nanobubbles have been detected using high-frequency ultrasound approaches, including 13–24 MHz imaging, as well as dual-frequency contrast imaging at 4 and 8 MHz, with detectability depending on acoustic pressure/mechanical index, shell mechanics, and pulse-sequence design.^82,83^ Shell properties are thought to play a major role in the behavior of MBs. Shells with lower elasticity, such as those composed of surfactants and phospholipids, are thought to be able to produce nonlinear signals at lower pressures, whereas stiffer shells may require a higher pressure to produce nonlinear signals.^66,84,85^ Polydispersity and concentration are also thought to play a major role in determining the behavior of MBs.

In practice, NBs are often driven off-resonance at clinical frequencies. As a result, detectability depends strongly on shell mechanics, pulse sequence design, and the usable pressure window. Section 5.2 examines how these factors constrain performance under clinical conditions and the stability–signal trade-offs that become important for translation. For a deeper treatment of contrast-agent acoustics, readers are referred to prior reviews,^86,87^ including NB-specific acoustics in a recent review by Wegierak et al.^22^

To summarize the foundational technical milestones discussed in this section, Figure 5 provides a selective timeline of key advances in NB identity/characterization, materials engineering, acoustic detectability, molecular targeting, and multimodal development.^28,31,62,76,88–92,92,94–97,97–102^

**Figure 5:**
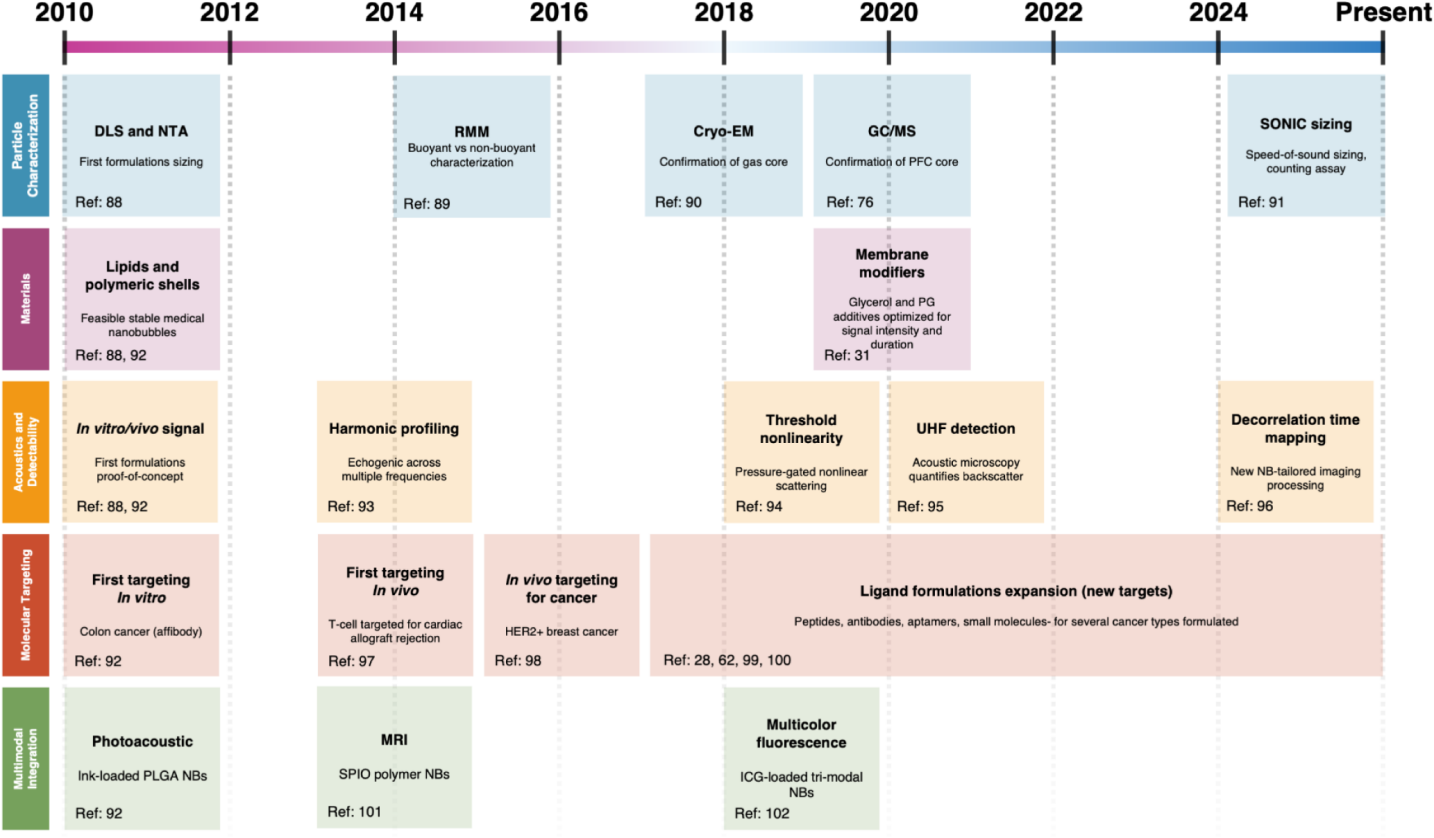
Milestones in NB contrast agent development (2010–present). A select timeline of the first reported instances (PubMed-indexed) of major advances in (1) particle characterization, (2) materials, (3) acoustics/detectability, (4) molecular targeting, and (5) multimodal integration. Each box marks the earliest report of the indicated capability (representative references shown). The multiyear ligand bar reflects a sustained expansion of targeting strategies rather than a single first-in-field event. This figure is a selective guide for historical context; these areas remain under active development. DLS: dynamic light scattering; NTA: nanoparticle tracking analysis; RMM: resonant mass measurement; Cryo-EM: cryogenic electron microscopy; GC/MS: gas chromatography–mass spectrometry; PFC: perfluorocarbon; UHF: ultrahigh frequency; HER2: human epidermal growth factor receptor 2; PLGA: poly­(lactic-*co*-glycolic acid); MRI: magnetic resonance imaging; ICG: indocyanine green.

## 4 Cancer Applications

NBs are an innovative approach to incorporating the principles of nanomedicine into ultrasound-based cancer imaging and therapy. The formation of NBs utilizes passive and active targeting strategies to enhance the tumor localization of the nanobubbles based on the principles of nanomedicine. Malignant tissues that are rapidly proliferating often develop abnormal vasculature that is very permeable, with abnormal endothelial junctions, fenestrations, and basement membranes. These characteristics can enhance the ability of the nanobubbles to extravasate beyond the vascular space into the tumor interstitial space. In addition, impaired lymphatic drainage can reduce the rate of efflux of the nanobubbles, thereby enhancing their retention in the tumor microenvironment (TME).^103,104^

NB tumor accumulation is not dictated solely by leaky vasculature and poor lymphatic drainage. Additional pathways—most notably active endothelial transport (e.g., transcytosis)^105^ and ultrasound-driven biophysical effects—can further promote vascular escape and retention.^22^ Because NBs are gas-filled and highly compressible, insonation can modulate their interactions with blood flow and the vessel wall, potentially increasing endothelial engagement and facilitating deeper vascular penetration. Together, these biological (cell-mediated uptake) and biophysical (acoustic/mechanical) contributions help shape NB distribution and persistence within the TME.

NBs can further be functionalized with the incorporation of targeting ligands into their shell, allowing binding to overexpressed tumor-specific or stromal receptors.^77^ This receptor binding can markedly increase their retention within tumors, despite targeted NBs’ extravasation from the vasculature being thought to be similar to that of nontargeted NBs.^28^ Direct intratumoral injection or cell-mediated transport via antigen-presenting cells are other approaches that can be used to achieve local delivery.^106^ Beyond that, other factors present in the clinical scenario have to be considered, such as interactions with red blood cells and the presence of plasma proteins, which have been shown to prolong NB circulation time and stabilize the acoustic signal.^107^

### 4.1 Imaging

#### 4.1.1 Untargeted Nanobubbles

Among the earliest and most accessible strategies for leveraging NBs in oncologic imaging is passive targeting, which capitalizes exclusively on increased vascular permeability in the tumor microvasculature (Figure 6B). Breast cancer has been a leading model for evaluating untargeted NBs, given the modality’s established role in diagnosis and biopsy guidance.^108^ In an orthotopic mouse model, Yang et al. showed that NBs incorporating IR-780 dye significantly improved tumor boundary delineation.^109^

**Figure 6:**
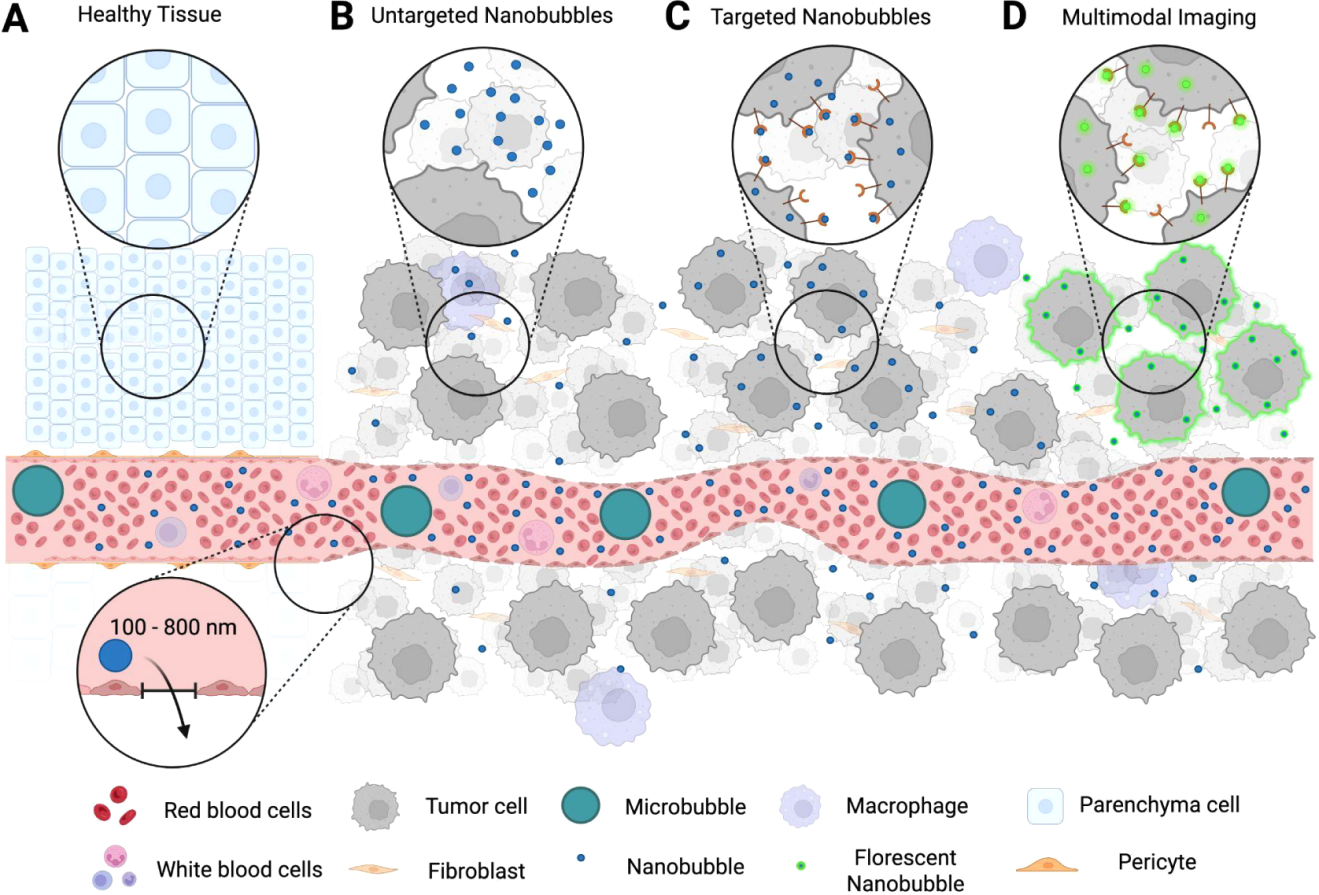
Schematic illustration of NB behavior in cancer imaging across different vascular and tissue contexts. A central blood vessel is shown running horizontally, with four scenarios: (A) Healthy tissue—normal vasculature with intact, continuous endothelial junctions prevents NB extravasation; both NBs (200–500 nm) and MBs (1–10 μm) remain intravascular. (B) Untargeted NBs—in tumor vasculature with fenestrated endothelium and abnormal architecture, NBs extravasate passively into the extracellular matrix (ECM) via the enhanced permeability and retention (EPR) effect, whereas MBs remain confined to the lumen. (C) Targeted NBs—surface functionalization with ligands (e.g., antibodies, peptides, small molecules) enables binding to overexpressed tumor or stromal receptors; some NBs undergo receptor-mediated endocytosis, further extending tissue retention. MBs remain strictly intravascular. (D) Multimodal NBs—targeted NBs coloaded with secondary imaging reporters (e.g., fluorescent dyes, magnetic nanoparticles, photoacoustic agents) support hybrid imaging approaches, combining ultrasound with complementary modalities (e.g., US–fluorescence or US–MRI). Figure not to scale. Made with BioRender.

Beyond structural enhancement, untargeted NBs have been used to probe tumor biology. Liu et al. incorporated DiI–DiD fluorophores to support FRET-based detection of myeloperoxidase (MPO)-driven inflammation in tumor tissues.^79^ Wu et al. used DiI-labeled NBs in breast (BC4T1) and ovarian (OVCAR3) tumor models, reporting 4.5-fold and 22.7-fold higher CEUS signal intensities, respectively, compared to MBs. The increased tumor-to-background signal suggested greater extravascular accumulation and retention.^110^

Wu et al. compared untargeted NBs to Definity MBs in colorectal tumor-bearing mice. Although peak enhancement was similar, NBs demonstrated slower *in vivo* signal decay and 1.67-fold longer *in vitro* stability, supporting their entrapment in tumor tissue and prolonged imaging window.^93^ A summary of untargeted NB formulations investigated for preclinical cancer imaging is provided in Table 2.

**Table 2:** Summary of Untargeted Nanobubble Formulations Used for Cancer Imaging in Preclinical Studies^a^

cancer type	shell composition	gas core	NB synthesis method	NB size (nm)	*in vitro* model	*in vivo* model	ref
breast and ovarian adenocarcinoma	DPPC, DSPE-PEG(2000), IR-780 iodide, glycerol	C_3_F_8_	thin-film hydration, rotary evaporation, C_3_F_8_ purging, mechanical agitation	442 ± 48	MDA-MB-231 and BT-474 human breast cancer, SKOV3 human ovarian cancer	MDA-MB-231 subcutaneous xenografts in BALB/c nude mice	^109^
breast adenocarcinoma	HSPC, DSPE-PEG2000, DiI, DiD	C_3_F_8_	ethanol lipid film hydration, sonication, mechanical agitation	178	neutrophils (for MPO), 4T1-luc mouse breast cancer	4T1-luc subcutaneous xenografts in Balb/c mice	^79^
breast and ovarian adenocarcinoma	DPPC, DPPA, DPPE, mPEG-DSPE, Pluronic L10, DiI, glycerol	C_3_F_8_	lipid film hydration in PBS, pluronic L10 and glycerol, mechanical agitation, differential centrifugation	138 ± 43	phantom	4T1 (mouse breast adenocarcinoma) orthotopic breast tumor and OVCAR-3 (human ovarian adenocarcinoma) subcutaneous ovarian tumors in female BALBc/4j mice	^110^
breast adenocarcinoma	C22, DPPA, DPPE, DSPE-mPEG2000, glycerol	C_3_F_8_	lipid film hydration in PG, glycerol and PBS, mechanical agitation, differential centrifugation	160	phantom	MET1 (mouse breast carcinoma) subcutaneous xenografts in FVB/NHanHsd mice	^83^
colorectal cancer	DPPC, DPPE, DPPA, Pluronic L61, glycerol	C_3_F_8_	lipid film hydration, mechanical agitation	207 ± 74	–	LS174T (human colorectal adenocarcinoma) subcutaneous xenografts in NCRnu/nu mice	^93^

a Overview of untargeted NB formulations investigated for cancer imaging in preclinical models, detailing their physicochemical composition, synthesis methods, and application *in vitro* and *in vivo*. NB: nanobubble; MPO: myeloperoxidase; PEG: polyethylene glycol; DPPC: 1,2-dipalmitoyl-*sn*-glycero-3-phosphocholine; DPPA: 1,2-dipalmitoyl-*sn*-glycero-3-phosphate; DPPE: 1,2-dipalmitoyl-*sn*-glycero-3-phosphoethanolamine; DSPE: 1,2-distearoyl-*sn*-glycero-3-phosphoethanolamine; DSPE-mPEG2000: DSPE bearing methoxy-PEG (2 kDa); HSPC: hydrogenated soy phosphatidylcholine; Pluronic L10/L61: poly­(ethylene oxide)-poly­(propylene oxide) triblock copolymer surfactants; DiI: 1,1′-dioctadecyl-3,3,3′,3′-tetramethyl-indocarbocyanine; DiD: 1,1′-dioctadecyl-3,3,3′,3′-tetramethyl-indodicarbocyanine; IR-780: heptamethine near-infrared fluorophore; C_3_F_8_: perfluoropropane (octafluoropropane); PG: propylene glycol.

#### 4.1.2 Targeted Nanobubbles

While untargeted NBs can passively accumulate in tumors through vascular abnormalities such as increased permeability, this mechanism is inherently heterogeneous and often insufficient for precise cancer imaging.^111,112^ The degree of vascular permeability and retention varies widely—not only between tumor types but also across lesions within the same patient—shaped by factors such as vascular density, interstitial pressure, stromal composition, and regional differences in the TME.^113^ In poorly vascularized or fibrotic tumors, or those exhibiting elevated interstitial fluid pressure, NB penetration into tissue may be significantly reduced.^104,114,115^ Moreover, passive uptake lacks cellular specificity, increasing the risk of off-target accumulation in nonmalignant tissues with chronic inflammation—such as autoimmune pancreatitis or diabetes mellitus type 1—where vascular permeability is also elevated. Targeted NBs that can bind receptors on cancer cells or stromal components may be used to overcome these limitations (Figure 6C).^28,77,116^

Prostate cancer is a useful example of the limitations of relying on solely passive targeting. Prostate tumors often display nonuniform and suboptimal uptake mediated by enhanced permeability and retention (EPR), with nonuniform perfusion and interstitial pressure observed both across and within tumors.^117–119^ In addition, peripheral and nonhomogeneous uptake of radiolabeled nanocarriers has been noted in a variety of prostate cancer xenograft models, with nonhomogeneous CD31 staining, a hallmark of abnormal vascular development.^117^ These features are consistent with the dense stroma and anatomical constraints often seen in prostate tumors, including castration-resistant disease, where tumor vasculature may be hypoxic or otherwise compromised in its ability to support nanoparticle delivery.^120,121^ Furthermore, biochemical characteristics of the tumor microenvironment, such as elevated glutathione, may compromise the integrity of the nanocarriers themselves.^122^ These factors highlight the limitations of passive targeting in prostate cancer and support the rationale for the application of active targeting, where ligand–receptor interactions mediate binding to overexpressed tumor markers.^117,123^

One of the most extensively studied molecular targets in prostate cancer is the prostate-specific membrane antigen (PSMA), which is highly expressed in malignant epithelial cells and largely absent in normal prostate tissue. Multiple preclinical investigations have demonstrated the feasibility and added value of PSMA-targeted NBs for improving CEUS imaging in prostate cancer models. For example, Fan et al. developed lipid-shelled NBs functionalized with an anti-PSMA aptamer and showed that targeted NBs selectively bound to PSMA-positive C4-2 cells *in vitro*, labeling 54.3% ± 4.2% of cells, while exhibiting negligible binding to PSMA-negative PC-3 cells (0.5% ± 0.3%). In PSMA-positive C4-2 tumor xenografts, targeted NBs yielded significantly higher peak intensity, prolonged half-peak duration, and increased area under the curve (AUC) compared to untargeted NBs (*P* < 0.05), with no significant differences observed in PSMA-negative models.^99^ On the basis of this groundwork, Perera et al. and Wang et al. used PSMA-targeted lipid NBs in heterotopic (xenograft) and orthotopic PC3pip mice prostate cancer models, respectively. The results demonstrated rapid accumulation and preferential retention of PSMA-NBs, with ∼2-fold higher washout AUC compared with untargeted NBs and the clinically approved MB Lumason (Figure 7D). Notably, nearly 48% of the PSMA-NB maximal signal remained after systemic washout, vs 25% for untargeted NBs in burst–replenishment imaging studies. Histological analysis also showed penetration of targeted NBs beyond the tumor vasculature.^28,124^ Aside from PSMA, Zhao et al. reported a polymer-based approach using ICG-loaded nanobeads that target the gastrin-releasing peptide receptor (GRPR), another prostate cancer-relevant biomarker. In GRPR-expressing tumor models, the targeted nanobeads produced a ∼4-fold increase in photoacoustic signal and a ∼2.0–2.2-fold increase in ultrasound intensity within tumors compared with nontargeted controls, consistent with strong receptor-specificity *in vivo*.^78^

**Figure 7:**
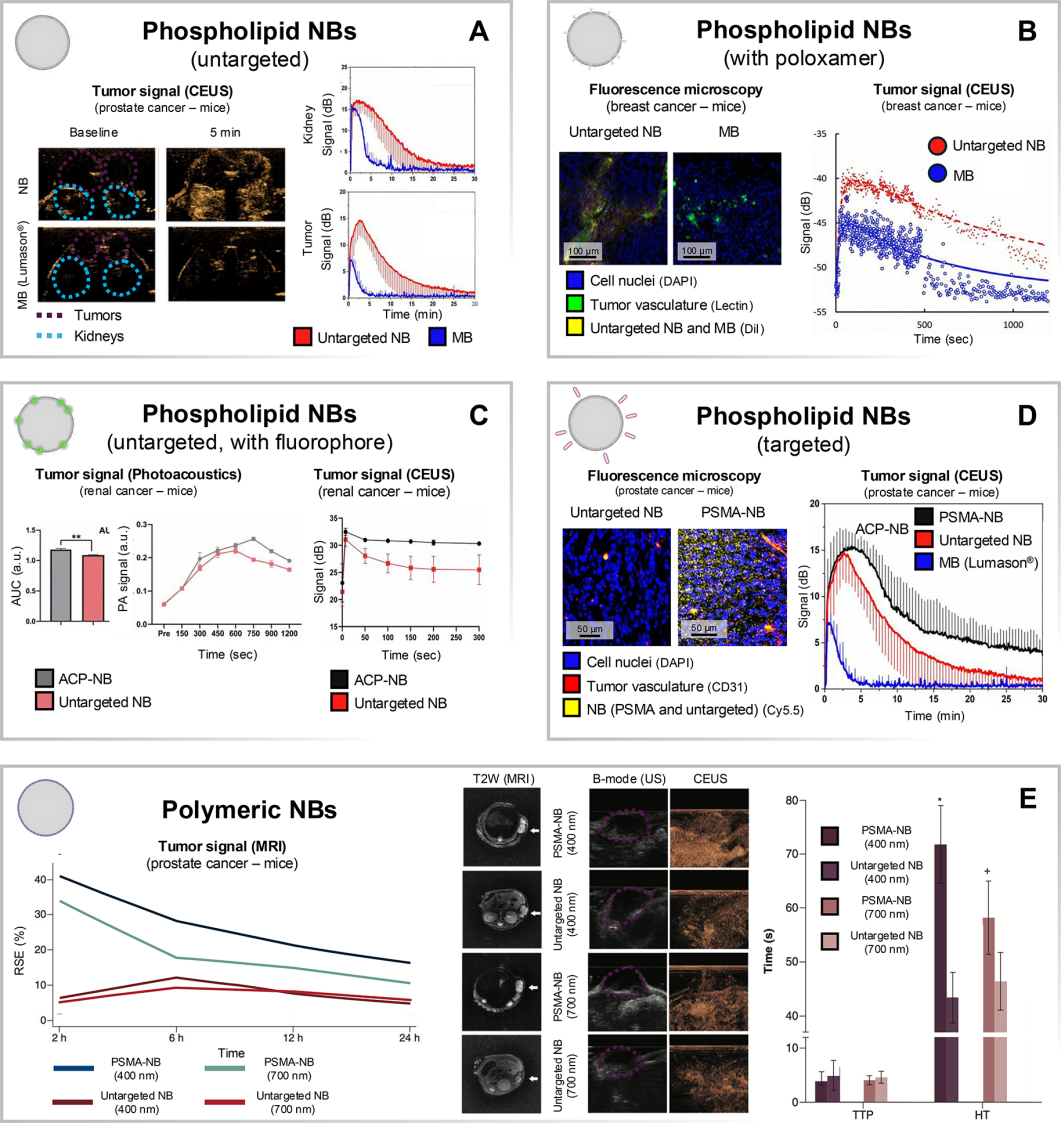
Representative studies across NB formulation classes. Each panel summarizes key findings from one representative paper per formulation class. (A) Phospholipid untargeted NBs: CEUS tumor and kidney signal kinetics vs MBs. Adapted with permission from Perera et al.^28^ Copyright 2020 Elsevier. (B) Phospholipid NBs with poloxamer: fluorescence microscopy (in OVCAR3 cells) and CEUS tumor signal (in BC4T1 cells) vs MBs. Adapted with permission from Wu et al.^110^ Copyright 2019 Elsevier. (C) Phospholipid NBs with fluorophores: CEUS tumor signal plus photoacoustic readouts of untargeted NBs and CA IX-targeted NBs in renal cell carcinoma mouse xenografts (786-O cells). Adapted with permission from Zhong et al.^116^ Copyright 2023 Taylor & Francis. (D) Phospholipid targeted NBs: fluorescence microscopy and CEUS tumor retention/kinetics vs untargeted, PSMA-targeted NBs and MBs in prostate carcinoma xenografts (PC3pip cells). Adapted with permission from Perera et al.^28^ Copyright 2020 Elsevier. (E) Polymeric NBs: tumor imaging kinetics with MRI integration alongside ultrasound/CEUS metrics of PSMA-targeted SPION NBs in prostate carcinoma xenografts (LNCaP cells). Adapted with permission from Zhu et al.^129^ Copyright 2020 Taylor & Francis. Colors were standardized across panels (e.g., MBs in blue; untargeted NBs in red). Panels were adapted (redrawn/reformatted) from the original publications. ACP-NB: CA IX-targeted polypeptide NBs. PSMA-NB: prostate-specific membrane antigen-targeted NBs. MRI: Magnetic resonance imaging. T2W: T2-weighted imaging. TTP: Time to peak. HT: half-life (time to reach 50% signal). RSE: Relative signal enhancement.

Beyond prostate cancer, targeted NBs have been explored in a variety of other malignancies, demonstrating the versatility of this platform across different tumor phenotypes and molecular targets. In preclinical breast cancer models, Du et al. used C_3_F_8_-filled poly­(lactic-*co*-glycolic acid) (PLGA) NBs functionalized with antibodies against human epidermal growth factor receptor 2 (HER2)—a transmembrane tyrosine kinase receptor overexpressed in aggressive breast carcinomas—and vascular endothelial growth factor receptor 2 (VEGFR2), a key regulator of tumor angiogenesis. The dual-targeted NBs showed significantly higher ultrasound signal intensity compared with the single-targeted and nontargeted NBs (*P* < 0.01), with enhanced tumor accumulation and prolonged imaging time.^77^ Another study by Johansen et al. used lipid NBs targeting protein tyrosine phosphatase mu (PTPμ), a cell adhesion molecule involved in tumor cell–cell interactions and invasion. *In vivo* imaging of tumor-bearing mice showed that PTPμ-targeted NBs produced >35% greater signal enhancement compared with scrambled NBs, with faster onset and sustained signal intensity throughout the 30-s imaging time window.^27^ For the study of lactoferrin (Lf), a naturally occurring iron-binding glycoprotein, the target of the NBs was the Lf receptor, LRP1, which is overexpressed in gliomas, breast, and ovarian cancers. In nude mouse models, Lf-targeted polymeric NBs (PAEEP-PLLA) showed significantly higher cellular uptake in LRP1-positive C6 glioma cells and produced strong and persistent signals across the entire tumor, significantly outperforming the clinical agent Lumason, which only provided peripheral tumor imaging because of the agent’s limited extravasation.^32^

For the study of papillary thyroid carcinoma, Xie et al. used lipid NBs functionalized with anti-BRAFV600E, targeting the oncogenic mutation of the BRAF gene, which determines aggressive cancer and poor patient prognosis. *In vivo* imaging of tumor-bearing mice showed a significantly higher peak intensity of the NBs in the BRAFV600E mutant PTC tumor compared with the untargeted NBs (11.45 ± 3.08 dB vs 1.22 ± 0.33 dB; *P* < 0.001), thus proving the specificity of the binding of the antibody with the target antigen.^125^ A summary of the different types of targeted NB formulations used for cancer imaging in animal models is provided in Table 3.

**Table 3:** Summary of Targeted Nanobubble Formulations Used for Cancer Imaging in Preclinical Studies^a^

cancer type	target molecule	targeting mechanism	shell composition	gas core	NB synthesis method	NB size (nm)	*in vitro* model	*in vivo* model	ref
prostate adenocarcinoma	PSMA	small molecule (DSPE-PEG-PSMA-1)	DBPC, DPPA, DPPE DSPE-mPEG 2000, DSPE-PEG-PSMA-1, PG and glycerol	C_3_F_8_	lipid film hydration with heating and sonication, mechanical agitation, differential centrifugation	277 ± 11	PC-3pip (PSMA+) and PC3flu (PSMA−) human prostate adenocarcinoma	PC-3pip and PC-3flu subcutaneous xenografts in male athymic nude mice	^28^
prostate adenocarcinoma	PSMA	monoclonal antibody (biotin–avidin)	DPPC, Bio-DSPE, DPPA, glycerol	C_3_F_8_	mechanical agitation, differential centrifugation, avidin–biotin-PSMA antibody conjugation	644 ± 55	LNCaP and C4-2 human prostate cancer	LNCaP and C4-2 xenografts in nude mice	^100^
prostate adenocarcinoma	PSMA	small molecule (DSPE-PEG-PSMA-1)	DBPC, DPPA, DPPE DSPE-mPEG 2000, DSPE-PEG-PSMA-1, PG and glycerol	C_3_F_8_	lipid film hydration with heating and sonication, mechanical agitation,differential centrifugation	277 ± 11	PC-3pip (PSMA+) and PC3flu (PSMA-) human prostate adenocarcinoma	PC-3pip and PC-3flu orthotopic tumors in male athymic Balb/c nude mice	^124^
prostate adenocarcinoma	GRPR	antibody (thiol-maleimide conjugated)	PLGA (80%), PLGA-PEG (10%), PLGA-PEG-Mal (10%), PVA, ICG	room air	double emulsion (water-in-oil-in-water), followed by solvent evaporation and lyophilization	322 ± 115	PC-3-GFP and DU145-GFP human prostate adenocarcinoma	PC3-GFP and DU145-GFP subcutaneous xenografts in nu/nu male mice	^78^
prostate adenocarcinoma	PSMA	peptide (CQKHHNYLC via EDC-NHS)	polypeptide-PLGA, SPIONs, PVA	SF_6_	double emulsion with NH_4_HCO_3_ and SPIONs, followed by lyophilization, SF_6_ purging and mechanical agitation	400 and 700	LNCaP and PC-3 human prostate adenocarcinoma	LNCaP and PC-3 subcutaneous xenografts in male BALB/cAnN-Foxn1nu/nu mice	^129^
prostate adenocarcinoma	PSMA	aptamer (A10-3.2, EDC-NHS conjugation)	DPPC, DPPA, DPPE, DPPG, PEG-2000, DSPE-PEG2000-COOH, glycerin	C_3_F_8_	lipid hydration and mechanical agitation, EDC/NHS coupling of A10-3.2 aptamer	519 ± 74	C4-2 and PC-3 human prostate adenocarcinoma	C4-2 and PC-3 subcutaneous xenografts in male BALB/c-nu nude mice	^99^
breast adenocarcinoma and angiosarcoma	HER2, VEGFR2	dual monoclonal antibodies (EDC/NHS-conjugated FITC-anti-HER2 and PE-anti-VEGFR2)	PLGA, camphor, PVA	C_3_F_8_	oil-in-water emulsion, solvent evaporation, lyophilization, antibody conjugation via EDC/NHS	230 ± 58	SKBR3 (HER2+) and MDA-MB-231 (HER2−) human breast adenocarcinoma, SVR (VEGFR2+) mouse angiosarcoma, 4T1 (VEGFR2−) mouse breast adenocarcinoma	SKBR3 subcutaneous xenografts in BALB/c nude mice	^77^
colorectal cancer	TAG-72	antibody (HuCC49ΔCH2 conjugated via NHS-biotin–streptavidin)	PLGA	room air	double emulsion with Texas Red dye; EDC/NHS and biotin–streptavidin conjugation	268 ± 110	LS174T human colorectal adenocarcinoma	–	^92^
renal cell carcinoma and cervical cancer	CA IX	nanobody (anti-G250, biotin–streptavidin coupling)	DPPC, DPPE, DPPA, DPPG, DSPE-PEG2000-biotin, glycerol	C_3_F_8_	membrane hydration, mechanical agitation, differential centrifugation	446 ± 43	786-O (G250+ human RCC), ACHN (G250– RCC), HeLa (G250+ cervical cancer)	786-O, ACHN, and HeLa subcutaneous xenografts in BALB/c-nu mice	^26^
glioblastoma	PTPμ	peptide (SBK2, DSPE-PEG conjugation)	DBPC, DPPE, DPPA, DSPE-mPEG2000, DSPE-PEG-SBK2	C_3_F_8_	lipid hydration and mechanical agitation	248 ± 85	–	LN-229 (human glioblastoma) subcutaneous xenografts in NCr-nu/nu mice	^27^
renal cell carcinoma	CA IX	peptide (ACP, biotinylated; coupled via streptavidin to biotin-PEG-lipid)	DPPC, DPPE, DPPG, DPPA, DSPE-PEG2000-Biotin, ICG dye	C_3_F_8_	filming rehydration, mechanical agitation, differential centrifugation	475 ± 48	786-O (CA IX+ human RCC), ACHN (CA IX– RCC)	786-O and ACHN subcutaneous xenografts in BALB/c-nu mice	^116^
renal cell carcinoma	CA IX	peptide (AGN, biotinylated; coupled via streptavidin to biotin-PEG-lipid)	DPPC, DPPE, DPPG, DPPA, DSPE-PEG2000-Biotin, ICG dye	C_3_F_8_	filming rehydration, mechanical agitation, differential centrifugation	411 ± 6	786-O (G250+ RCC), ACHN (G250– RCC)	orthotopic PDX models in NPG mice	^130^
glioma	LRP1	lactoferrin (Lf) ligand covalently conjugated via PEG linker (Mal–PEG–NHS chemistry)	PAEEP-PLLA amphiphilic block copolymer	C_5_F_12_	O_1_/O_2_/W double emulsion and solvent evaporation; Lf conjugation via thiolation and PEG-maleimide coupling	328 ± 5	C6 glioma (LRP1+), ECV304 (LRP1−)	C6 glioma subcutaneous xenografts in BALB/c nude mice	^32^
B-cell lymphoma (BL and CLL/SLL)	CD20 (B-cell marker)	rituximab (anti-CD20 antibody) conjugated via periodate oxidation/reductive amination to chitosan shell	chitosan	C_5_F_12_	ethanol injection, ultra-Turrax homogenization; dropwise addition of chitosan; antibody conjugation and antagomiR17 encapsulation	400	BJAB (Burkitt lymphoma, miR17-high), MEC-1 (CLL/SLL, miR17-low), THP-1 (human macrophages)	intraperitoneal BJAB (BL) or MEC-1 (CLL/SLL) xenografts in SCID mice	^131^
papillary thyroid carcinoma	BRAF^V600E^	biotin–streptavidin conjugation of biotinylated BRAFV600E monoclonal antibody to biotin-DSPE-PEG2000	HSPC, DSPG, DSPE-PEG2000, Biotin-DSPE-PEG2000	C_3_F_8_	thin-film hydration; sonication; antibody conjugation	173 ± 8	B-CPAP (human PTC cell line) transfected with BRAF^V600E^ plasmid	B-CPAP subcutaneous xenografts in BALB/c nude mice	^125^

a Overview of targeted NB formulations for preclinical cancer imaging, including targeting ligands, conjugation strategies, and evaluation across *in vitro* and *in vivo* models. NB: nanobubble; GFP: green fluorescent protein; PDX: patient-derived xenograft; SCID: severe combined immunodeficiency; PSMA: prostate-specific membrane antigen; GRPR: gastrin-releasing peptide receptor; HER2: human epidermal growth-factor receptor-2; VEGFR2: vascular endothelial growth-factor receptor-2; TAG-72: tumor-associated glycoprotein-72; CA IX (G250): carbonic anhydrase IX; PTPμ: receptor-type protein tyrosine phosphatase-μ; LRP1: low-density lipoprotein receptor-related protein-1; CD20: cluster of differentiation 20; BRAFV600E: valine-to-glutamate activating mutation in B-RAF; PLGA: poly­(lactic-*co*-glycolic acid); PVA: poly­(vinyl alcohol); PEG: polyethylene glycol; Mal: maleimide; EDC: 1-ethyl-3-(3-(dimethylamino)­propyl)­carbodiimide; NHS: *N*-hydroxysuccinimide; SPION: superparamagnetic iron-oxide nanoparticle; ICG: indocyanine green; DBPC: 1,2-dibehenoyl-*sn*-glycero-3-phosphocholine; DPPC: 1,2-dipalmitoyl-*sn*-glycero-3-phosphocholine; DPPE: 1,2-dipalmitoyl-*sn*-glycero-3-phosphoethanolamine; DPPA: 1,2-dipalmitoyl-*sn*-glycero-3-phosphate; DPPG: 1,2-dipalmitoyl-*sn*-glycero-3-phospho-(1′-rac-glycerol); DSPG: 1,2-distearoyl-*sn*-glycero-3-phospho-glycerol; DSPE: 1,2-distearoyl-*sn*-glycero-3-phosphoethanolamine; DSPE-mPEG2000: DSPE bearing methoxy-PEG (2 kDa); HSPC: hydrogenated soy phosphatidylcholine; PAEEP-PLLA: poly­(amide-amine-ethylene phosphate)-*block*-poly­(l-lactic acid); C_3_F_8_: perfluoropropane (octafluoropropane); C_5_F_12_: perfluoropentane (dodecafluoropentane); SF_6_: sulfur hexafluoride; NH_4_HCO_3_: ammonium bicarbonate (thermal porogen); PG: propylene glycol; Lf: lactoferrin; Camphor: sublimable porogen for polymeric particles; ACP: CA IX-binding peptide; AGN: CA IX-binding peptide; SBK2: PTPμ-binding peptide; A10-3.2: PSMA-binding RNA aptamer.

#### 4.1.3 Multimodal Imaging

Multimodal imaging, which is defined as the combination of two or more imaging techniques in a single procedure, has emerged as a cornerstone in precision medicine in the field of oncology and interventional radiology. The advantage of using multimodal imaging is the ability of the clinician to utilize the strength of different imaging techniques in a single procedure.^126^ Techniques like MRI–US fusion are now standard in guiding prostate biopsies, improving the detection of clinically significant cancers (median 33.3% vs 23.6%) while reducing the number of cores required (median 9.2 vs 37.1).^127^ Similarly, PET/CT and SPECT/CT have long paired anatomical and molecular imaging to enhance lesion localization and characterization.^128^ NBs are increasingly being adapted for such integrated approaches. While targeted NBs inherently support molecular ultrasound, the addition of fluorophores, photoacoustic agents, or magnetic components enables cross-platform imaging capabilities (Figure 6D).

Yang et al. developed NBs loaded with IR-780, enabling both CEUS and near-infrared fluorescence (NIRF) imaging. In the context of breast cancer, these nanoscale bubbles (NBs) have been shown to enhance the visualization of the tumor margins and achieve longer-term fluorescence accumulation within the tumor for up to 24 h, thereby extending the spatial resolution that could be obtained with conventional ultrasound imaging alone.^109^ ICG-loaded nanobubbles that are capable of generating robust ultrasound (US) and photoacoustic (PA) signals were reported by Zhao et al.^109^ When used in GRPR-positive prostate tumors, these NBs demonstrated more than 3-fold specificity in targeting compared with untargeted controls. Fluorescence imaging was used to confirm the specificity of targeting. Ref 78 reported the use of superparamagnetic iron oxide nanoparticles (SPIONs) in polymeric nanobubbles targeted against the PSMA receptor, resulting in a pronounced effect in reducing the signal in LNCaP tumors (Figure 7E). The MRI signal persisted for longer than that obtained with ultrasound, suggesting that some fragments of the NBs with SPIONs had become entrapped within the tissues.^129^

## 5 Challenges and Future Directions

Many preclinical studies have pointed out the promising applications of NBs in molecular imaging, multidetection, and initial theranostics. However, some challenges must be addressed in order to move NBs into clinical trials. From a chemistry and materials science point of view, these issues define a translational pathway in which early formulation and manufacturing decisions directly determine eventual clinical usability. The current section identifies the main gaps in the field, not solely related to the identity and characterization of the NBs, but also regarding their ability to translate well from model organisms to humans, and the demands expected to be addressed toward the usability of these particles in clinical settings. To frame these translational gaps across formulation classes, Figure 8 provides a best-in-class benchmark scorecard summarizing how current tumor-imaging NB studies report (or omit) key parameters related to imaging sequences, *in vivo* acoustics, persistence, particle characterization rigor, and model maturity.

**Figure 8:**
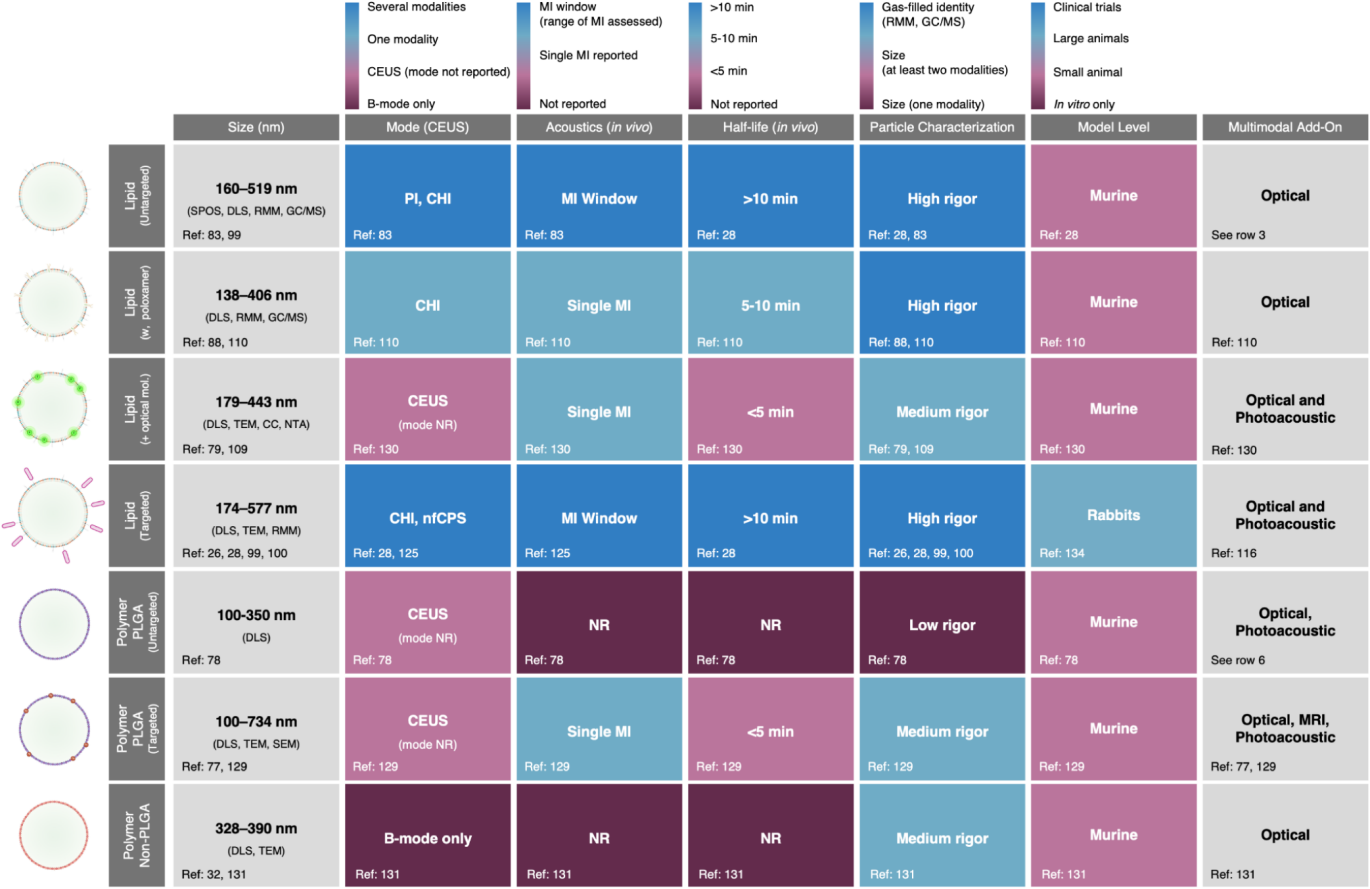
NB benchmark scorecard for tumor imaging. Heatmap summarizing best-in-class reporting and performance for NB formulations used for tumor imaging, restricted to the same studies included in Tables 2 and 3. Rows group studies by formulation class. For each formulation class (row), entries reflect the highest level achieved across papers in that class (“best-in-class”). Thus, if any study in a class (1) confirmed gas-filled identity (e.g., GC/MS or RMM), that row is scored as high rigor for characterization; or (2) tested an MI range/window (vs a single MI), that evidence is used for the acoustics score. Size reports the minimum-to-maximum mean diameters (smallest and largest reported averages) across studies in that class and lists all characterization methods used across those studies. CEUS: contrast-enhanced ultrasound; MI: mechanical index; NR: not reported; SPOS: single-particle optical sizing; DLS: dynamic light scattering; RMM: resonant mass measurement; NTA: nanoparticle tracking analysis; GC/MS: gas chromatography–mass spectrometry; TEM: transmission electron microscopy; SEM: scanning electron microscopy; CHI: contrast harmonic imaging; PI: pulse inversion; nfCPS: nonlinear fundamental contrast pulse sequence.

Other types of nanoscale UCAs, such as nanodroplets and gas vesicles, are also explored in preclinical research but are not discussed at length in this narrative review. Nanodroplets are made of liquid perfluorocarbons, which, via an acoustic phase change, vaporize to create echogenic MBs, while certain types are also capable of repeated activation via recondensation.^132^ On the other hand, gas vesicles are proteinaceous, empty nanostructures, which are biosynthesized in microorganisms and are genetically customizable and acoustically responsive.^133^ Both exhibit potential for molecular targeting and other applications of translational medicine, but extensive challenges persist in their activation, immune interactions, and regulatory hurdles.^132,133^ Examples of alternative innovations in nanoscale sonography are bound to the narrative outline to focus on specific concerns in relation to the development of NBs.^26,28,77,78,83,89,99,100,110,116,125,129–131,134^

### 5.1 Nanobubble Identity and Characterization

Translation begins with strict characterization of the particles. Across the NB literature, inconsistent or single-modality characterization remains a frequent source of irreproducible biological outcomes and, in some cases, overstated performance. For NBs, “nanosized” is not synonymous with “gas-filled”. Rigorous validation must establish a gas core, demonstrate acoustic activity consistent with a bubble, and distinguish NBs from adjacent nanoscale particles (e.g., liposomes, lipid nanoparticles, micelles, nanodroplets).

The techniques that were proposed to validate the presence of a gas core are as follows RMM, which can distinguish between buoyant and nonbuoyant particles;^73^ headspace gas GC-MS for the quantification of the volume of the gas core;^135^ pressure collapse assays that can evaluate the effect of pressure collapse on the signal and/or the number of particles;^136^ cryo-electron microscopy for the visualization of the presence of a gas core;^137^ and compressibility measurements that can evaluate the presence of the gas core at the assembly level.^91^ The key point to note is that these techniques are not interchangeable. For example, optical sizing can only provide information on the hydrodynamic diameter of the NBs and not on the composition and acoustic activity can also be caused by the presence of a small number of microbubbles. As a result, NB claims supported only by size metrics—or by a single technique—remain difficult to interpret and compare across studies.

The use of only size measurements is also not advisable because common sizing methods have built-in biases that can obscure even small, but highly echogenic, subpopulations. DLS provides intensity-weighted distributions that may be skewed by larger scatterers and cannot distinguish gas-filled bubbles from lipid or polymer particles.^136,138^ Likewise, NTA reports size and concentration but does not confirm the composition of the tracked objects, and it may underestimate gas-containing subpopulations.^135^ This becomes even more consequential in polydisperse samples. The presence of MBs (∼1–10 μm) can contribute backscatter and harmonic signal.^136,138,139^ MBs can be beneficial as they can provide clear vascular delineation, yet their presence can lead to signal attenuation and confound the interpretation of extravascular NB signal.

For these reasons, the field would benefit from a minimum, fit-for-purpose validation framework that links physical identity to functional performance. At a baseline, this includes reporting size distributions (with method limitations stated), together with a buoyancy-sensitive measure of particle concentration when feasible (e.g., RMM),^73^ at least one independent line of gas-core evidence (e.g., GC-MS, pressure-collapse behavior, or compressibility),^91,135,136^ and standardized acoustic characterization that specifies frequency, pressure or mechanical index, and pulse sequence while quantifying backscatter and harmonic output.^66,140^ Because translation ultimately depends on behavior in physiologic environments, stability should be assessed under relevant conditions (e.g., serum at 37 °C) using acoustic readouts in addition to particle sizing.^76,141^ In parallel, minimum reporting should include formulation details (shell components and amounts, gas type, PEG molecular weight/density, and ligand chemistry/density where applicable), preparation and purification steps, acoustic testing parameters, storage and handling conditions, and some assessment of batch-to-batch variability.

Several interpretive guardrails are especially important for avoiding misattribution. Claims of NB-mediated signal should therefore include controls and analyses that support accurate attribution of the measured acoustic response to the intended particle population and biological compartment (intravascular vs extravascular).^73,136^ NB stability (in terms of signal decay rates under constant insonation) should be examined *in vitro* using ultrasound parameters identical to those applied *in vivo*. For claims of molecular targeting, controls must be included to verify the specificity of interaction, e.g., using scrambled ligand formulations, receptor-negative animal models, and competitive blocking agents.^142^ Detection of the targeted nanobubble (NB) agents must be done using ultrasound and be validated *in vitro* and *in vivo*, as appropriate, with radiology/pathology support to verify target specificity. The overall series of procedures ensures that results obtained accurately represent the true biological activity of the formulation of interest, thereby providing a better foundation for model comparison and the advancement of preclinical development. To help define the minimum, fit-for-purpose characterization procedures, Figure 9 presents a practical workflow linking orthogonal gas-core validation, sizing/concentration measurements, standardized acoustic testing, and *in vivo* PK/PD, biodistribution, and safety studies for novel NB formulations.

**Figure 9:**
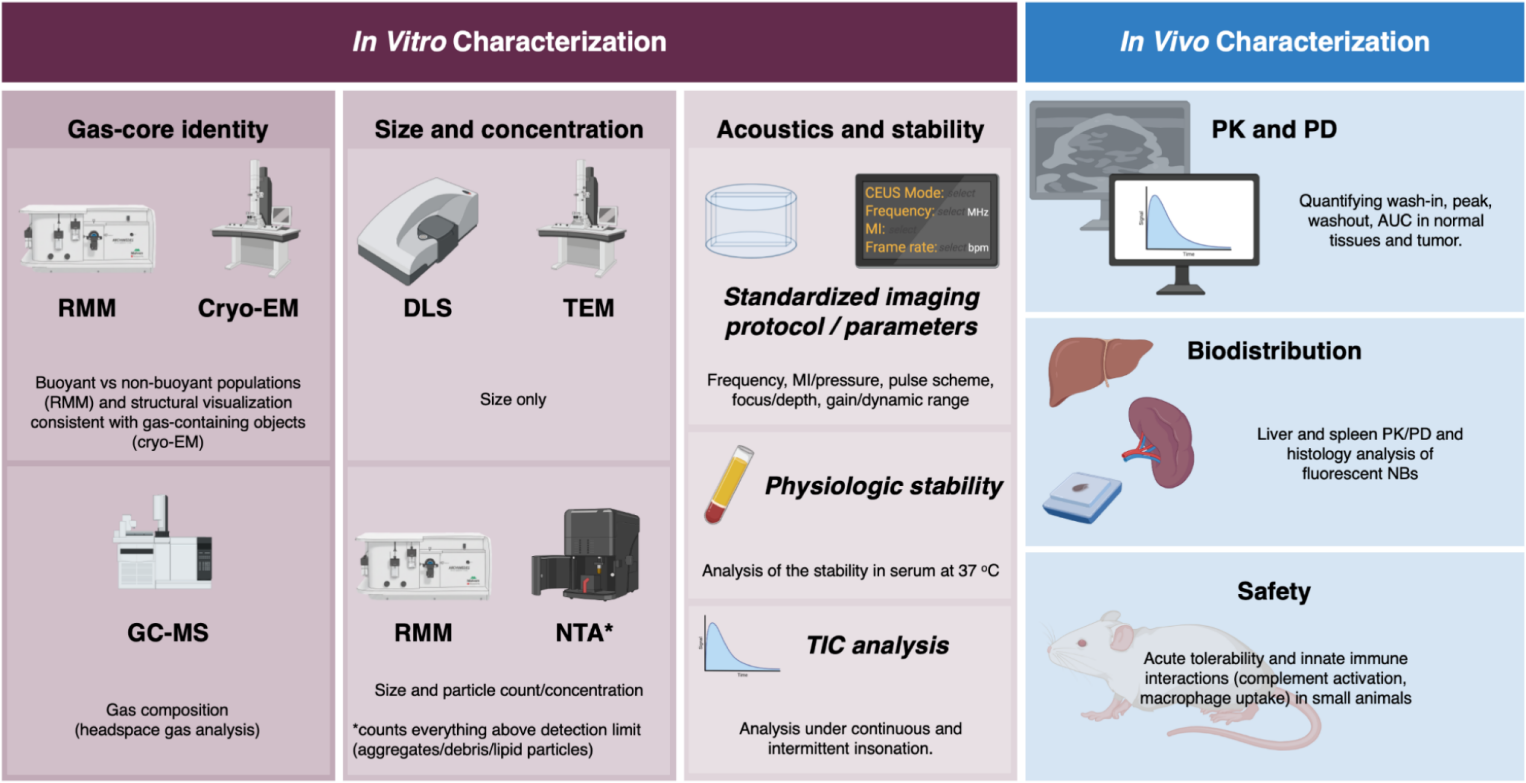
Fit-for-purpose workflow for characterizing new ultrasound bubble formulations. The *in vitro* panel integrates gas-core confirmation, size/concentration measurements, and acoustics/stability testing using standardized imaging parameters, physiologic stability, and TIC analysis. The *in vivo* panel summarizes PK/PD kinetics in normal tissues and tumor, biodistribution/clearance assessment, and an initial safety screen focused on acute tolerability and innate immune interactions. RMM: resonant mass measurement; cryo-EM: cryogenic electron microscopy; GC-MS: gas chromatography–mass spectrometry; DLS: dynamic light scattering; TEM: transmission electron microscopy; NTA: nanoparticle tracking analysis; MI: mechanical index; TIC: time–intensity curve; PK: pharmacokinetics; PD: pharmacodynamics. Made with BioRender.

### 5.2 Stability and Acoustic Performance

An effective equilibrium between structural stability and acoustic detection is critical for the successful translation of NBs. The balance of these two critical factors is dominated by the nanoscale gas physics and chemistry. Specifically, the strong dependence of the Laplace pressure on the small bubble radius, often reaching several atmospheric values, facilitates the dissolution of gases^66^ and reduces the longevity of the uncoated bubbles relative to that of MBs.^143^ Stabilization of the bubbles can be accomplished by the use of low-solubility perfluorocarbon gases and low gas-permeability shells, typically by the use of cross-linked shells. In these formulations, the echogenicity of the bubbles can be sustained for long periods of time *in vitro* and several days *in vivo* following intratumoral injection.^144,145^ In practice, the stability of the bubbles can be enhanced by the use of stiffer shells, typically by the application of cross-linking agents or by the addition of protective coatings.^146,147^ These modifications, however, may cause changes in the dynamics of the oscillation and the phase of nonlinear behavior, typically buckling, and thus affect the harmonic response in a pressure- and frequency-dependent fashion. More compliant and possibly permeable shells may enhance the nonlinear response of the bubbles, but these formulations may be subject to the adverse effects of shear force-induced gas leakage and the presence of serum proteins,^31,145,148^ potentially causing instability and resulting in the phenomenon of Ostwald ripening.^148^

These physicochemical limitations manifest as formulation-dependent stability-signal trade-offs that need to be managed in the design of the NB. In general, improvements in gas retention capability are associated with decreased permeability of the shells, which is often concomitant with increased cross-linking and rigidity; however, this may require higher activation pressures to induce nonlinear scattering. The threshold activation pressures required for the detection of nonlinear scatter for 200 nm diameter particles are frequency-dependent, ranging from 123 to 245 kPa for more compliant shells to 588–710 kPa for stiffer shells.^66^ Surface modification by PEGylation may improve circulation persistence by reducing opsonization; however, it may also reduce echogenicity and limit access of targeting ligands, while simultaneously increasing circulation persistence.^149^ The addition of targeting ligands may improve binding; however, it may simultaneously increase the surface charge, induce aggregation, and alter the viscoelasticity of the shells in a way that reduces oscillations.^148,149^ The addition of therapeutic molecules may further complicate the situation, as the drugs or dyes may interfere with the lipid bilayer structure, permeability, and resonance; in some instances, the signal may decay more rapidly in drug-loaded shells compared to empty shells.^66,150^ Furthermore, smaller bubble sizes may improve the chances of gaining access to the extravascular space; however, smaller scatterers may require higher concentrations of the agent, higher frequency and pressure, or more sensitive detection systems. However, in the frequency- and pressure-dependent range above the threshold activation pressures, in the harmonic and superharmonic range, and in a volume-matched comparison (equal gas volume), it has been demonstrated that submicrometer-sized agents may have higher overall nonlinear signal compared to larger particles despite the decreased per agent scatter.^151,152^

Acoustic performance is further constrained by clinical imaging conditions. Although nonlinear scattering from NBs has been observed at clinically relevant frequencies (≈2.5–8 MHz),^94^ estimated theoretical resonance frequencies for nanoscale bubbles can be orders of magnitude higher (≈50–200 MHz), implying that most clinical systems drive NBs off-resonance.^24,143,153^ In principle, this mismatch limits harmonic generation relative to MBs imaged closer to resonance and increases the importance of shell mechanics and pulse sequence design in determining detectability. However, the periodic buckling and rupture of lipid-shelled bubbles can markedly enhance acoustic scattering and is regarded as the key mechanism underlying the efficacy of NB formulations despite being far off resonance.^24,66,151,154^ Cavitation thresholds are similarly formulation- and frequency-dependent; inertial cavitation has been reported under certain conditions (e.g., albumin-stabilized NBs at ≈3.3–5.4 MHz),^155^ yet standardized operating windows that distinguish safe diagnostic imaging from therapeutic cavitation remain poorly defined across NB classes. Importantly, many encouraging preclinical demonstrations rely on high-frequency transducers (≈12–40 MHz), where NB responses are favorable, but such results do not necessarily predict equivalent performance at the lower frequencies (≈1–5 MHz) required for deep-tissue oncologic imaging.^94,153^ As a result, validation at clinically relevant frequencies and scanner configurations remains a central barrier to adoption.

To move translation of this field forward, evidence needs to be provided beyond static sizing metrics, including functional characterization in a clinically relevant context. This includes, as a minimum, acoustic response curves (backscatter and harmonic content), cavitation thresholds as a function of pressure over a range of clinically relevant frequencies, and stability time courses in serum at 37 °C using acoustic rather than size-based metrics. It should also include resilience testing under injection- and flow-relevant mechanical stresses, since handling and shear can alter both size distributions and acoustic activity. Across the literature, it remains difficult to find well-characterized data sets that link NB stability under physiological conditions to consistent acoustic performance on clinically relevant imaging platforms, underscoring the gap between promising proof-of-concept studies and the robustness required for translation.

### 5.3 Biointerface, Pharmacology, and Safety

A major translational challenge is that NB pharmacokinetics (PK) and pharmacodynamics (PD) remain incompletely defined; however, because the *in vivo* behavior of gas-core UCAs is broadly governed by the same biointerface factors that shape MB performance—shell chemistry, surface charge, ligand interactions, and the evolving protein corona^156–158^ —much of the established MB intravascular PK/PD framework is likely directly applicable to NBs. In contrast to MBs, whose circulation within the blood pool and clearance are relatively well defined, NB pharmacokinetics appear more complex, NBs likely share the same fundamental intravascular circulation and clearance pathways in intact, healthy vasculature, but can require additional characterization in settings of vascular permeability and disease-associated transport beyond the blood pool. Circulation studies have reported prolonged half-life and a “second-wave” phenomenon, in which a second signal peak appears minutes after the initial bolus.^93,159^ The reasons for this biphasic pattern are not yet fully understood, but may include redistribution from a tissue compartment, delayed capillary transit, or interactions with circulating cells.

Clearance mechanisms for NBs in the intravascular space likely parallel those established for MB UCAs, particularly given that many MB formulations contain substantial NB-range subpopulations. In MBs, the gas component is eliminated predominantly by dissolution with pulmonary exhalation,^160,161^ while the shell is handled largely by the mononuclear phagocyte system with uptake in the liver and spleen and natural lipid metabolism.^162,163^ Accordingly, differences reported for NBs are best interpreted in the context of shared biointerface-driven RES processing, with additional complexity arising mainly when disease-associated permeability enables transport beyond the blood pool.^164,165^ This becomes even more complex for targeted NBs, where receptor-mediated endocytosis can introduce an additional pathway for uptake and retention.^166^ This can prolong the signal, and multiple studies have demonstrated formulation- and target-dependent differences in *in vivo* kinetics and tissue retention using dynamic CEUS and complementary biodistribution readouts. For example, NB pharmacokinetic profiles and organ-level kinetics have been quantified across tumors and major organs,^97,110,135^ and several reports support enhanced tumor retention/extravasation relative to MB comparators when assessed by fluorescence/histology or concurrent visual-acoustic tracking.^110,124,167^ While these findings are encouraging, continued standardization across formulations—particularly with respect to shell composition, polydispersity, surface charge, and ligand density—will further clarify how opsonization and cell interactions shape whole-body distribution and clearance.

Safety data remain limited, but they suggest clear formulation-dependent effects. In animal models, neutral lipid-shelled NBs have generally appeared well tolerated, with no evident hepatotoxicity or nephrotoxicity.^168^ In contrast, some cationic lipid formulations have been associated with severe hepatotoxicity.^168^ Targeted NBs have also been reported to increase splenic macrophage activity, which likely relates to clearance.^164^ These observations are consistent with established principles for clinically used MB UCAs—where biointerface and shell composition shape immune interactions and RES uptake—and are particularly relevant given that many MB preparations include substantial NB-range subpopulations.^169,170^ As these agents advance, standardized, MB-aligned safety and immunology panels will help enable cross-study comparability without implying fundamentally different requirements from existing UCA development frameworks.

### 5.4 Model Selection and Translational Scale-Up

So far, the majority of preclinical work on NBs has been conducted *in vitro* or in small animal models such as mice and rats. Although these types of studies can provide a preliminary assessment of the feasibility and basic mechanisms, they are not reliable in making predictions about the translation of the results to the clinic in humans. A systematic review conducted by Perel et al. showed that the results from animal model studies were in agreement with the results from the corresponding human clinical trial only about 50%.^171^ This discrepancy is not only because of scaling laws but also because of other reasons. Although mice share more than 90% of their genome with humans,^172^ they also possess very different physiologies. For example, the basal metabolic rate in mice is higher, and the heart rate is about eight times faster than that in humans.^173^

Ultrasound-related issues add another level of complexity to the translation from the mouse model. Small-animal imaging studies are conducted while the animals are anesthetized, while in the clinic, patients are awake and spontaneously breathing. Anesthesia also affects the cardiovascular system, which in turn affects the circulation time of the UCAs.^174^ More importantly, the type of anesthesia used, either medical air or pure oxygen, reduces the half-life of the MB by more than 30%, an effect that is not considered in the translation from the mouse model.^175^ At the same time, the most commonly used small-animal models of cancer are not representative of the most common forms of cancer in the clinic. The most commonly used models are derived from mouse tumor cell lines, which are not clinically relevant, or require profound immunosuppression for the growth of the tumor from human cell lines. Moreover, the subcutaneous model does not reflect the tumor microenvironment and pressures that are present in the clinic.^176^

Collectively, these constraints inform the strategic progression toward larger animal models that more accurately reflect the physiology, tumor model, and imaging conditions relevant to the human population. Each of the rabbit, pig, and canine models offers unique advantages, with the pig model more accurately reflecting the cardiovascular and immunologic characteristics relevant to the human population (80% immune homology compared with 10% in mice).^177^ The canine model offers unique advantages for prostate and cardiovascular models, with these models more accurately reflecting the anatomy and electrophysiology of the human population.^178–180^ The rabbit model offers an attractive intermediate model that more accurately reflects the cardiovascular dynamics in the human population while offering logistical and cost advantages.^181,182^ The determination of the performance characteristics of NBs in these more accurate models will be critical in determining the relevant dosing, imaging, and safety profiles prior to first-in-human studies.

### 5.5 Manufacturing, Quality Control, and Regulation

Aside from the biological feasibility, the translation of NBs for the clinic also relies on the capability for consistent and large-scale production, the existence of relevant characterization techniques for the release of the product, and the preparation of a regulatory package that meets current chemistry, manufacturing, and controls (CMC) requirements. Currently, the major body of research on nanobubbles appears to be focused on the proof-of-concept and early formulations rather than clinically relevant developments. The process conditions are also significant, especially because they can have an impact on properties that are directly relevant to the clinic, such as the average diameter, gas content, surface chemistry, and acoustic potency.

One important practical note is that the manufacturing process scale and the state of the art in preclinical research today may not easily scale to commercial production. While extrusion is routinely used to manufacture liposomes at liter scales and can yield well-defined size distributions,^183^ its translation to NB production will primarily require confirming that size control and acoustic potency are maintained under scaled process conditions.^71^ Probe sonication can produce high encapsulation efficiency of the gas if the process is highly controlled for factors like temperature; however, the process is inherently sensitive to the energy levels and the design of the probes.^184^ Microfluidics provides excellent control over the particle size and concentration and can improve yields beyond standard laboratory scale; however, the main limiting factor remains the throughput.^185^ The mechanical agitation process is straightforward and can produce high concentrations; however, the main issue remains the production of particles with wide size distributions.^71,186^ In these processes, several factors could very well turn out to be critical process parameters: these include the mixing energy and the mixing time, the process of gas exchange, the process of purification, and the process of fill and finish.

Establishing a CMC path therefore requires defining critical quality attributes (CQAs) and linking them to clinically meaningful performance. Importantly, NB CMC and quality control can and should be built on the same established principles used for clinically approved MB UCAs (identity, strength/potency, purity/impurities, and stability), with fit-for-purpose extensions only where NB formulations introduce additional features (e.g., nanoscale distributions or targeting ligands). For NBs, CQAs will likely include size distribution and polydispersity, particle (or bubble) number concentration, gas-core fraction or an orthogonal surrogate, shell composition consistency, and surface chemistry parameters such as PEG density and ligand presentation.^71,184–186^ Many of these are conceptually analogous to attributes already controlled for MB products, but may be measured with different methods depending on size regime and formulation. For example, size can be reported by DLS or NTA, which are commonly used in nanoparticle characterization, but neither confirms a gas core; buoyancy-sensitive measurements (e.g., RMM) can help differentiate buoyant from nonbuoyant populations, yet are not universally applied.^73^ Similarly, ligand density and surface chemistry are often reported at a qualitative or semiquantitative level, and additional standardization of assays suitable for release testing would strengthen comparability across studies. Accordingly, a central translational need is to define practical, standardized assays—aligned with existing MB CMC expectations—that can be used to demonstrate lot-to-lot consistency and support comparability across manufacturing changes.

A related unmet need is a functional potency assay suitable for release testing. Because the therapeutic and diagnostic value of NBs is inseparable from their acoustic behavior, CMC development will likely require an acoustic potency metric—such as nonlinear backscatter amplitude or activation thresholds measured under standardized frequency, pressure (or mechanical index), and temperature conditions—that correlates with *in vivo* performance.^71,184^ Stability-indicating assays should likewise incorporate acoustic readouts (rather than size alone) after storage, handling, and exposure to physiologic media, since degradation that minimally affects hydrodynamic size can still reduce echogenicity or shift activation behavior.^184^ In addition, MB contamination deserves explicit consideration in release testing because even a small micron-scale fraction can dominate the acoustic signal, while specific thresholds may be formulation- and application-dependent, studies that quantify and control larger bubble populations are more readily interpreted and compared.^185^

Sterility and endotoxin control represent additional constraints that are often underemphasized in preclinical NB reports but will be unavoidable for clinical translation. Sterile filtration at pore sizes commonly used for injectables may be incompatible with certain NB size distributions or may compromise bubble integrity, making aseptic manufacturing strategies—using sterile components and validated assembly—particularly relevant.^187,188^ The feasibility and impact of terminal sterilization approaches remain uncertain for many NB formulations, and systematic recovery and potency testing after sterilization steps are rarely reported.^187,188^ Endotoxin control is similarly critical: how standard assays (e.g., LAL-based methods) perform with gas-core colloids and surfactant-rich formulations, and what process controls are required to reliably meet injectable limits, warrant explicit evaluation as CMC programs mature.^187,188^

From a regulatory standpoint, early translation will likely be facilitated by positioning NBs initially as diagnostic UCAs, where there is precedent for gas-core formulations and well-defined safety expectations from clinically approved MB UCAs.^189^ However, NB-specific guidance is limited, and IND-enabling packages will still need to address core elements including GLP toxicology, biodistribution and clearance, immunological interactions (including complement activation and macrophage engagement), and hemocompatibility, with careful attention to formulation-specific risks.^187,188^ Comparability will also be central: because NBs are sensitive to processing and surface chemistry, even modest formulation changes (e.g., PEG density, ligand conjugation strategy, gas selection) may necessitate bridging studies unless CQAs and potency assays are sufficiently predictive.^187,188^ Finally, codevelopment with imaging systems should be considered part of productization rather than an afterthought. Standardized, disease-specific, scanner-relevant protocols—and, ideally, vendor-compatible presets and instructions for use—will be necessary to ensure that acoustic performance demonstrated in controlled research settings can be reproduced reliably in clinical practice.^189^

### 5.6 Clinical Translation Roadmap

Integration of NBs into the clinic will require overcoming the technical challenges outlined above and building a body of evidence that is acceptable for translation. In order to clearly demarcate a structured framework for such development, we hereby introduce a stage-gated translational development process that entails identity and chemistry, manufacturing, and controls readiness through compatibility with the clinical protocol and first-in-human study, as depicted in Figure 10. In the near term, priority should be given to generating robust data in larger animal models that more closely approximate human physiology and tumor biology and also performing a careful assessment of NB pharmacokinetics and toxicity in clinically relevant models. These studies will be necessary to meet regulatory expectations, including frameworks outlined by the FDA for nonclinical safety testing, and to support the initiation of early human trials.^190^ At the same time, NB performance will need to be compatible with the capabilities and constraints of ultrasound devices already integrated into the clinical practice to ensure that the NB technology can be incorporated into the daily clinical activities.

**Figure 10:**
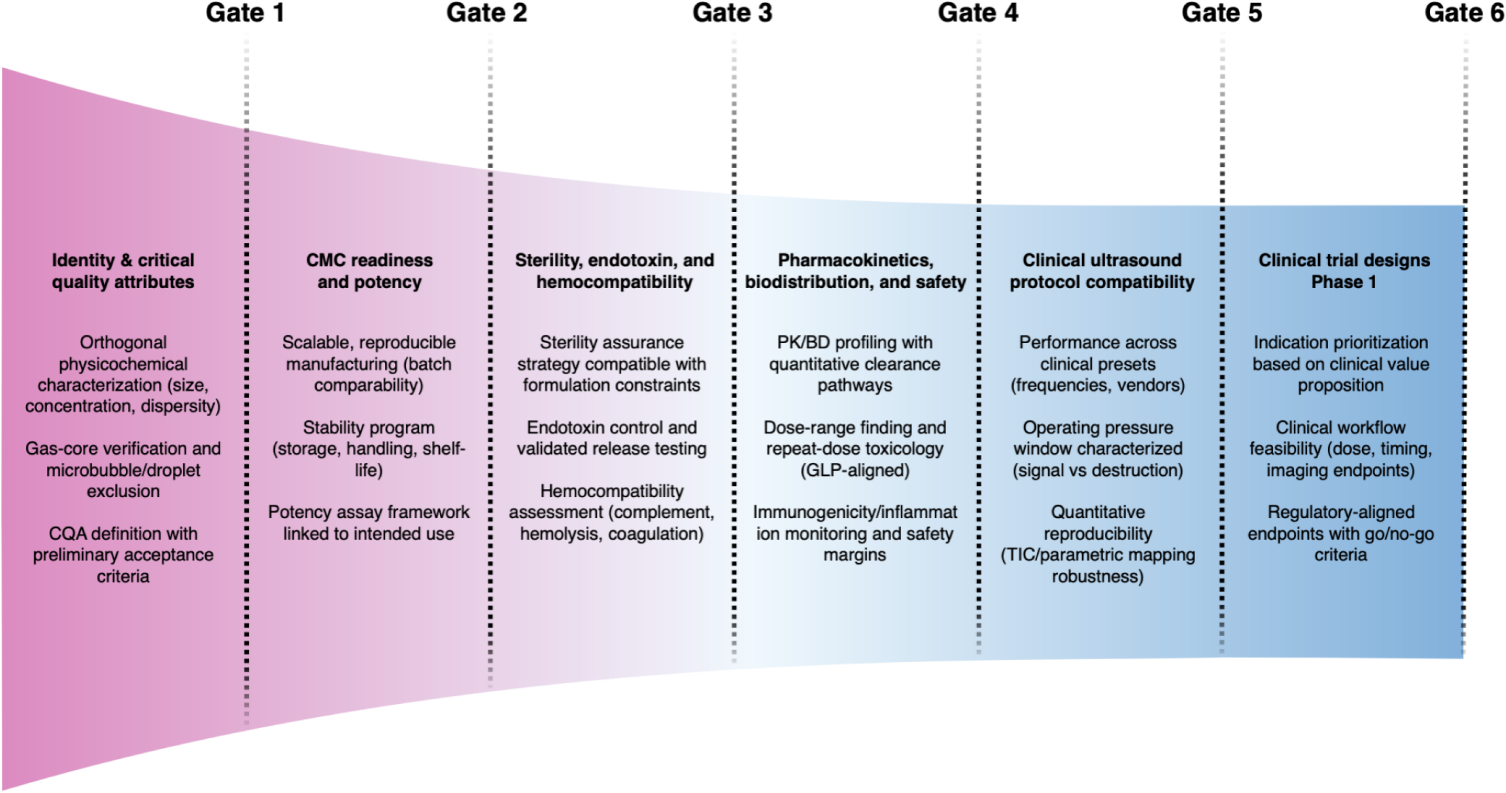
A stage-gated approach to the translation of NB contrast agents is described, comprising six gates to be passed sequentially. Gate 1 relates to identity verification and the definition of critical quality attributes. Gate 2 incorporates the readiness of chemistry, manufacturing, and controls (CMC) as well as the development of potency assays. Gate 3 relates to sterility and endotoxin testing together with hemocompatibility assessments. Gate 4 relates to pharmacokinetic and biodistribution profiling, together with the safety assessment based on Good Laboratory Practice (GLP) requirements. Gate 5 considers compatibility with the clinical ultrasound presets as well as the quantitative reproducibility of measurements. Gate 6 relates to the stratification of the first-in-human clinical use based on the definition of clinical trials. Progress from one gate to the next depends on the achievement of specified acceptance criteria and batch-to-batch consistency.

A potential entry point for clinical use of NBs is to introduce them first as purely diagnostic contrast agents in malignancies where conventional ultrasound is already incorporated into the clinical diagnostic workflow, such as breast or prostate cancer. This strategy could build on the established clinical safety record of current MB UCAs,^191^ positioning NBs as a next-generation platform that retains the favorable safety profile of existing UCAs while adding the potential for molecular targeting and evaluation of the extravascular compartment. Early clinical studies would ideally focus on indications where the additional information is likely to change management without requiring major changes to current imaging workflows.

In the longer term, NBs might extend their application from diagnostics to related clinical oncology activities. Having proven their effectiveness and safety in a diagnostic setting, NBs might then be used as an additional treatment tool and offer further benefit to patients with cancer. They might improve image-guided targeted biopsy by focal direction of biopsies in areas of more aggressive tumors and improve oncologic therapies such as thermal ablation and focused ultrasound treatments. Further opportunities might then arise as new techniques are developed, such as the real-time assessment of the treatment response and the activation of NBs by ultrasound waves for drug delivery to a specific target area.

## Supplementary Material



## Notes

Google Patents and PubMed search strategies for Figure 1 intellectual-property and publication trend analyses are available online (PDF)

## Vocabulary

Nanobubbles (NBs): Submicron (≈200–500 nm) gas-filled, shell-stabilized particles that generate ultrasound contrast, can extravasate into tumor tissue, and be functionalized for imaging or therapy.
Microbubbles (MBs): Gas-filled contrast agents (≈1–10 μm) confined to the vasculature that enhance ultrasound signal through nonlinear oscillations but generally cannot extravasate.
Contrast-Enhanced Ultrasound (CEUS): Ultrasound imaging technique that uses gas-filled agents (MBs or NBs) to amplify backscatter and enable vascular and, with NBs, extravascular/molecular assessments.
Tumor Microenvironment (TME): The cellular and acellular milieu surrounding tumor cells—vasculature, stroma, immune cells, matrix—governing nanoparticle transport, retention, and biointeractions.
Molecular Targeting: Ligand-mediated binding of contrast agents to overexpressed receptors (e.g., PSMA, HER2), improving tumor specificity, signal persistence, and diagnostic accuracy.
Theranostics: Integrated diagnostic-therapeutic strategies in which NBs provide molecular imaging and, upon acoustic activation, enable localized drug/gene delivery or mechanochemical tumor effects.
