# Supplementary material for: New Frontiers in Contrast-Enhanced Ultrasound for Cancer Imaging
Source: ACS Nano. 2026 May 12;20(20):14327–50. doi: 10.1021/acsnano.5c17992 (PMC13218053; doi:10.1021/acsnano.5c17992)
Supplement: Supplementary file 1 [file nn5c17992_si_001.pdf]

New Frontiers in Contrast-Enhanced Ultrasound for Cancer Imaging

Felipe Matias Berg[1,2], Michaela Briana Cooley[3], Theresa Kosmides[3], Laura E. Chen[3], Ronaldo Hueb Baroni[2*], Agata A. Exner[1,3*]

Authors affiliations:

[1] Department of Radiology, Case Western Reserve University, Cleveland, OH, 44106, United States

[2] Department of Radiology, Hospital Israelita Albert Einstein, São Paulo, SP, 05652-900, Brazil

[3] Department of Biomedical Engineering, Case Western Reserve University, Cleveland, OH, 44106, United States

Supplementary File

Correspondence to:
Prof. Ronaldo H. Baroni
Department of Radiology, Hospital Israelita Albert Einstein
Av. Albert Einstein, 627, São Paulo, SP, 05653-000, Brazil
rbaroni@einstein.br
or
Prof. Agata A. Exner
Department of Radiology and Department of Biomedical Engineering, Case Western Reserve University,
10900 Euclid Avenue, Cleveland, OH, 44106, USA
agata.exner@case.edu
*Co-last authors

Search Strategy In Google Patents in Figure 1

This is the search strategy used to generate the bar graph of patents about nanobubbles for medical imaging applications and exclude nanobubbles used for other purposes (mainly water treatment)

TI=(nanobubbl* OR "nano bubble" OR "nano bubbles" OR nano-bubbl* OR "nanoscale bubble" OR "nanoscale bubbles" OR "submicron bubble" OR "submicron bubbles" OR "sub-micron bubble" OR "sub-micron bubbles" OR "ultrafine bubble" OR "ultrafine bubbles" OR "ultra-fine bubble" OR "ultra-fine bubbles")

AND TAC=(ultrasound OR CEUS OR "ultrasound imaging" OR "ultrasound contrast" OR "contrast enhanced ultrasound" OR echogenic* OR sonication OR echograph* OR sonograph*)

AND TAC=(medical OR patient OR "in vivo" OR diagnos* OR imaging OR therap* OR treatment OR "contrast agent" OR "drug delivery" OR "nucleic acid" OR gene OR tumor OR cancer)

AND TAC=-wastewater AND TAC=-"water treatment" AND TAC=-sewage AND TAC=-flotation AND TAC=-detergent AND TAC=-cleaning AND TAC=-agriculture AND TAC=-soil AND TAC=-irrigation AND TAC=-aquaculture AND TAC=-concrete AND TAC=-cement AND TAC=-petroleum AND TAC=-"oil recovery" AND TI=-"generat*" AND TAC=-"water" AND TAC=-"waste" AND TI=-"liquid" AND TAC=-"fertiliz*"

Search Strategy In PubMed papers in Figure 1

This is the search strategy used to generate the bar graph of PubMed files about nanobubbles for medical imaging applications since 2009 and exclude nanobubbles used for other purposes (mainly water treatment)

(nanobubbl*[ti] OR "nano bubble"[ti] OR "nano bubbles"[ti] OR nano-bubbl*[ti] OR
"nanoscale bubble"[ti] OR "nanoscale bubbles"[ti] OR
submicron bubble*[ti] OR "sub-micron bubble"[ti] OR "sub-micron bubbles"[ti] OR
ultrafine bubble*[ti] OR "ultra-fine bubble"[ti] OR "ultra-fine bubbles"[ti])
AND
(ultrasound[tiab] OR CEUS[tiab] OR "contrast enhanced ultrasound"[tiab] OR
"ultrasound imaging"[tiab] OR "ultrasound contrast"[tiab] OR
echogenic*[tiab] OR sonograph*[tiab] OR echograph*[tiab] OR sonication[tiab])
AND
(medical[tiab] OR patient*[tiab] OR "in vivo"[tiab] OR diagnos*[tiab] OR imaging[tiab] OR
therap*[tiab] OR treatment[tiab] OR "contrast agent*"[tiab] OR
"drug delivery"[tiab] OR gene[tiab] OR "nucleic acid"[tiab] OR tumor*[tiab] OR cancer*[tiab])
NOT
(wastewater[tiab] OR sewage[tiab] OR flotation[tiab] OR detergent*[tiab] OR cleaning[tiab] OR
agriculture[tiab] OR soil[tiab] OR irrigation[tiab] OR aquaculture[tiab] OR
concrete[tiab] OR cement[tiab] OR petroleum[tiab] OR "oil recovery"[tiab] OR
fertiliz*[tiab] OR "water treatment"[tiab])
